# Alteration of empathy-related behaviors in two strains of mice expressing mutations of the α5 cholinergic nicotinic receptor subunit

**DOI:** 10.1016/j.isci.2026.117950

**Published:** 2026-10-17

**Authors:** Léa Tochon, Cécile Pagèze, Nadia Henkous, Jean-Louis Guillou, Vincent David

**Affiliations:** 1 University of Bordeaux, INCIA, 33000 Bordeaux, France

**Keywords:** affective state discrimination, prosocial-like behavior, alpha5 nicotinic receptors, alcohol use disord

## Abstract

A single nucleotide polymorphism (SNP) of the alpha5 subunit of nicotinic acetylcholine receptors (nAChRs) increases human risk for nicotine dependence, alcohol use disorders (AUDs), and schizophrenia. Here, we used transgenic mice carrying either this SNP (rs16969968; α5SNP/α5KI) or a Chrna5 knockout (α5KO) to examine the role of α5-containing nAChRs in affective state discrimination and prosocial, rescuing-like behaviors. α5KO mice failed to recognize a negative affective state in a familiar peer and displayed severely altered prosocial, empathy-related behaviors, at times assaulting distressed peers. In contrast, α5KI mice discriminated affective states normally and showed increased rescuing-like behavior. Effects of α5 mutations on affective state discrimination were sex stratified. These findings demonstrate the critical implication of α5∗nAChRs in affective state discrimination and prosocial-like behaviors, revealing sex-stratified patterns of these social-emotional deficits in α5KI and α5KO mice, which are reminiscent of the behavioral features of two characterized subtypes of AUD in humans.

## Highlights

• α5KO mice fail to discriminate a distressed from a neutral peer
• α5KO mice show reduced rescue-like behavior and increased aggression
• α5KI mice retain normal affective discrimination and rescue peers more
• α5KO and α5KI profiles parallel behavioral features of two AUD subtypes

## Graphical abstract

**Figure:**
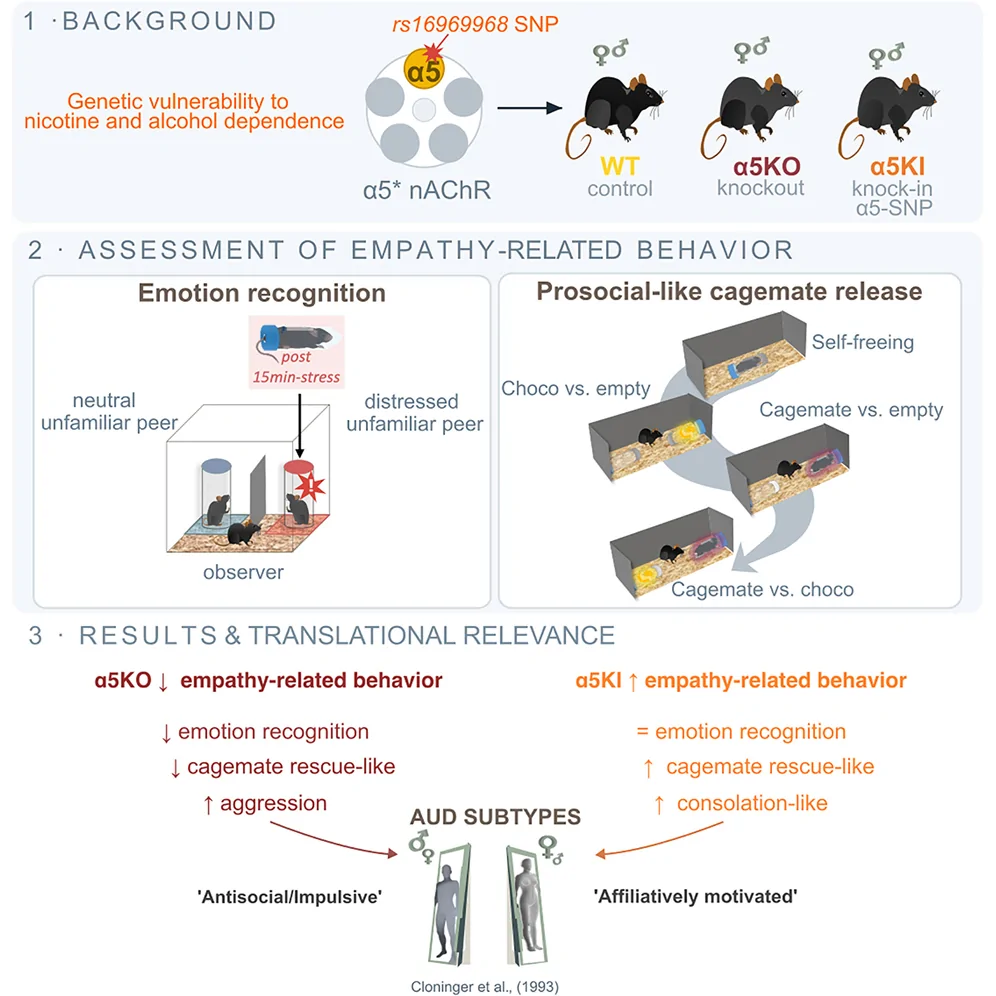

## Teaser

Biological sciences; Behavioral neuroscience; Cognitive neuroscience; Model organism

## Introduction

Human genome wide studies (GWAS) have reported a link between a single nucleotide polymorphism (SNP rs16969968) of the *CHRNA5* gene encoding for the α5 subunit of nicotinic acetylcholine receptors (nAChRs), and the likelihood of developing nicotine addiction, alcohol use disorder (AUD) and schizophrenia.^1^^,^^2^^,^^3^^,^^4^^,^^5^^,^^6^^,^^7^

The α5 SNP is a frequent missense variation replacing an aspartic acid with an asparagine (D398N), with homozygous carriers showing a 2-fold increased risk for smoking.^8^^,^^9^ Genetic manipulations of α5 containing nAChRs (α5∗nAChRs) in rodents have linked a null mutation or SNP of *Chrna5* with differences in alcohol self-administration and tolerance.^7^^,^^10^^,^^11^^,^^12^ Transgenic rats expressing the α5SNP through a genetic knock-in (α5KI) procedure consume more alcohol and exhibit increased relapse to alcohol seeking after abstinence.^13^ Consistently, we have demonstrated that both α5KI and α5KO mutant mice showed alcohol over-consumption for highly concentrated solutions, but display opposite anxiety-related behavior (hyper vs. hypo-anxious) and behavioral control (not impulsive vs. impulsive-like, respectively).^7^ We pointed out that these opposite phenotypes strongly evoke the two opposite human subtypes of AUD initially referred to as Type I and Type II based on different characteristics such as personality traits, biological sex, and age of onset.^14^^,^^15^ Cloninger’s *Type I* (“*Avoidance alcoholism”*) is attributed to middle-aged individuals who are prone to anxiety and who are thought to indulge alcohol in a “self-medicating” way to reduce stress and cope with their symptoms.^14^ At the other end of the continuum, Cloninger’s *Type II* (“*Sensation-seeking alcoholism”*), which has a male predominance, is characterized by impulsivity and sensation-seeking behavioral traits accompanied by antisocial behavior and displays of aggression; for this subtype, alcohol is suggested to be used to enhance their sense of thrill.^14^^,^^16^

In light of our previous data showing behavioral similarities between α5KI and α5KO mutant mice, the Cloninger’s avoidant (Type I) and sensation-seeking (Type II) AUD subtypes, respectively, we raise the hypothesis that α5 nicotinic mutants may offer a relevant basis for investigating behavioral analogies with AUD subtypes.^7^ However, one remaining issue about the relevance of these mouse models concerns their social-emotional profile. AUD is associated with difficulties in emotion processing and social cognition, including deficits in empathy-related processes such as facial emotion recognition.^17^^,^^18^^,^^19^^,^^20^^,^^21^^,^^22^^,^^23^^,^^24^^,^^25^^,^^26^ Empathy in its broad sense can be defined as an induction process that reflects the cognitive ability to perceive the emotional states of others and the affective tendency to experience emotions of others, which can be coupled with the motivation to care for their well-being and to help by acting prosocially (i.e., acting to benefit another).^27^^,^^28^

The difficulty to respond appropriately to another’s emotions can compromise interpersonal relationships, thereby contributing to the onset and/or the sustainment of alcohol-related problems but also interfere with recovery.^21^^,^^29^ However, the types of social-emotional impairment seem to differ across AUD subpopulations as observed in Cloninger’s Type I and II. The first are described as “eager to help others” and “sensitive to social cues” but seemed to misinterpret facial expressions of sadness and contempt.^14^^,^^30^ By contrast, the latter were found to overinterpret facial expressions of anger and disgust, showed a preponderance of anger mimicry and aggressive behaviors,^14^^,^^31^ which is associated with deficient affective empathy^32^ and is congruent with their high rate of comorbidity with antisocial personality disorder.^14^^,^^31^ There is therefore a crucial need for better characterization of emotional and social cognitive processes to understand the different motivations leading to alcohol abuse and to develop suitable interventions taking into account patients’ socio-emotional skills.^33^

In the past years, rodents have been increasingly used to study social capabilities considered as empathy-related features.^34^^,^^35^^,^^36^^,^^37^^,^^38^ New experimental protocols have been developed to assess their affective state discrimination abilities^39^^,^^40^^,^^41^^,^^42^^,^^43^ and prosocial motivation to help.^40^^,^^44^^,^^45^^,^^46^^,^^47^^,^^48^ Moreover, α5-nAChRs are expressed in key brain structures involved in social cognition and social-emotional processing such as the amygdala, prefrontal cortex,^49^^,^^50^ subiculum, ventral hippocampus,^51^^,^^52^ olfactory bulb, and hypothalamus.^53^^,^^54^ We thus investigated the functional impact of either the α5SNP or a null mutation of *Chrna5* on affective state discrimination abilities and prosocial-like, empathy-related behaviors in mice. We hypothesized that either α5KO and/or α5KI would perform differentially from WT mice on these social tasks, thus demonstrating the implication of α5-nAChRs in socio-emotional behaviors and potentially furthering the analogy between these mutant strains and the behavioral characteristics of two different AUD subtypes.^14^

## Results

### Emotion discrimination in α5KO and α5KI mice

We first assessed the ability of α5KO and α5SNP (=α5KI) transgenic mouse strains to discriminate between two unfamiliar mice (called demonstrators) in different affective states, i.e., either a state of psychological stress induced by 15 min of constraint inside a tube (SD), or a neutral state (ND). We found both strains to be sensitive to the presence of demonstrator mice inside the cylinders, as revealed by the time spent in the demonstrator zones during the test trial as compared to the three habituation trials in all groups (M + F; trial effect: F_(3,123)_ = 116.46, *p < 0.001,* partial *η*^2^ = 0.74, 95% CI [0.656, 0.787]*;* sex effect: F(1,41) = 2.81, *p* = 0.10; Figures 1A and 1D). When analyzed separately by sex, the trial effect remained highly significant in both males (F(3,63) = 55.54, *p* < 0 .001, partial *η*^2^ = 0.73, 95% CI [0.59,0.79]) and females (F(3,60) = 77.03, *p* < 0 .001, partial *η*^2^ = 0.79, 95% CI [0.68,0.84]), with no genotype main effect in either sex (males: F(2,21) = 0.44, *p* = 0.65; females: F(2,20) = 0.40, *p* = 0.68). Notably, the genotype × trial interaction was significant in males (F(6,63) = 3.27, *p* = 0.007) but not in females (F(6,60) = 0.88, *p* = 0.52), consistent with the more pronounced genotype-dependent impairment observed in males at “Test”. In WT mice, time spent in the SD zone relative to the ND zone was higher similarly for both sexes (t(17) = 3.14, *p* = 0.006, *d* = 0.74, 95% CI [0.175, 1.305]; M: t(8) = 1.38, *p* = 0.20; F: t(8) = 3.36, *p* = 0.01; Figures 1B and 1E). In contrast, α5KO mice (both male and female) appeared severely impaired in this task, spending an equivalent amount of time in each zone (M: t(7) = 0.406, *p* = 0.70; F: t(4) = 1.105, *p* = 0.331; Figures 1B and 1E). α5KI exhibited an intermediate performance. Interestingly, however, while male α5KI appeared severely impaired in this task (t(6) = 0.40, *p* = 0.70; Figure 1B), their female counterparts were still able to discriminate between SD and ND demonstrators (t(8) = 2.36, *p* = 0.046, *d* = 0.79, 95%CI [-0.106, 1.679]; Figure 1E).

**Figure 1:**
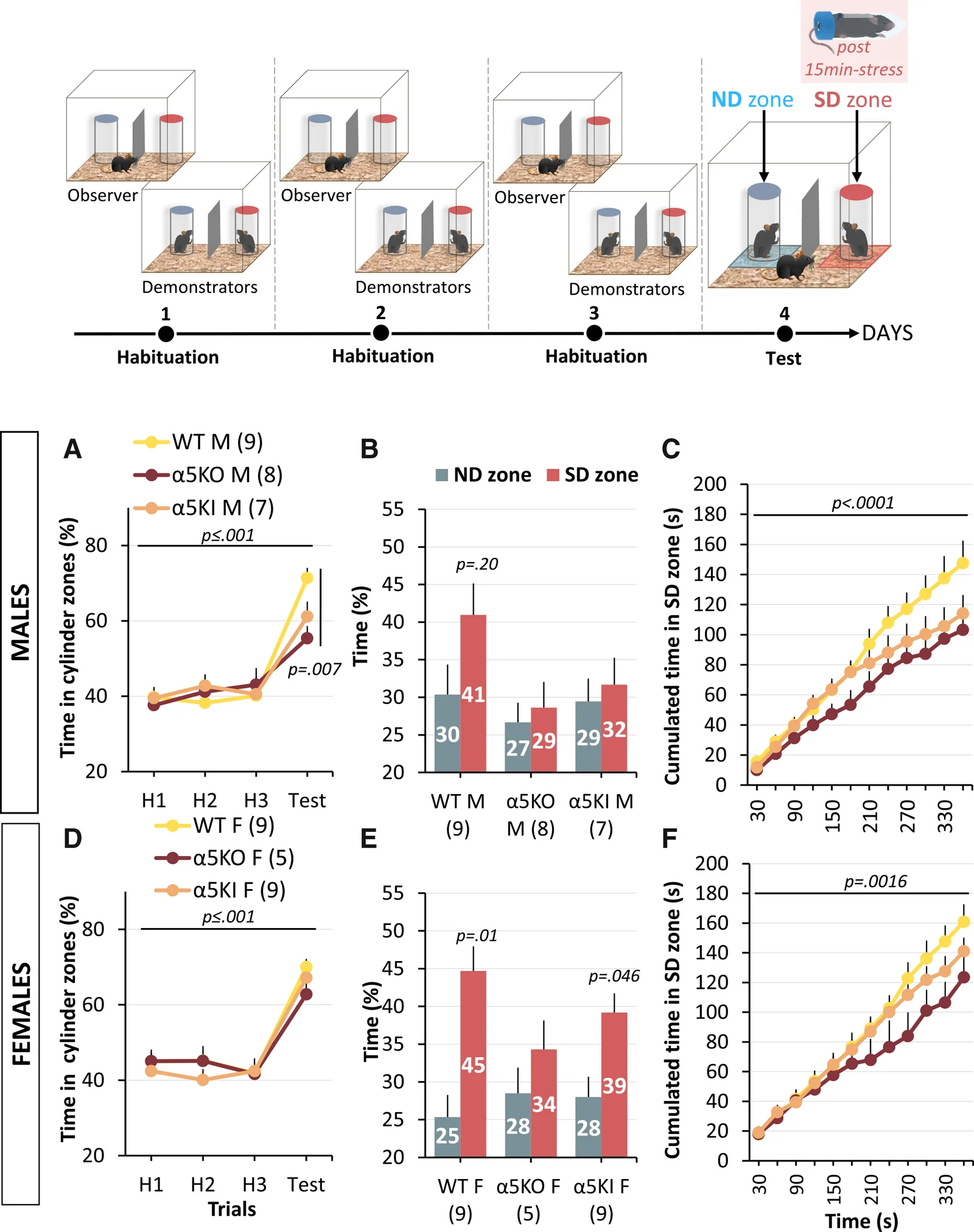
Genotype- and sex-stratified affective state discrimination deficits in α5 nicotinic mutant mice Top. Protocol of the Affective State Discrimination Task (ASDT). Mice were habituated over three trials (H1–H3) to two demonstrators, then tested on day 4 against a neutral demonstrator (ND, blue) and a demonstrator stressed by 15-min restraint (SD, red). (A and D) Time spent in the cylinder zones across trials, in males (A) and females (D). Both sexes spent more time near the cylinders at test vs. habituation (trial effect, RM-ANOVA: males *p* < 0 .001; females *p* < 0 .001). Genotype × trial interaction was significant in males (RM-ANOVA, *p* = 0.007) but not females (*p* = 0.52). (B and E) Time spent in the ND (blue) vs. SD (red) zone at test, in males (B) and females (E). WT mice discriminated SD from ND in both sexes (paired *t* test: males *p* = 0.20, females *p* = 0.01). α5KO mice failed to discriminate in either sex (males *p* = 0.70; females *p* = 0.33). α5KI males failed to discriminate (*p* = 0.70), whereas α5KI females still discriminated (*p* = 0.046). (C and F) Cumulative time in the SD zone across the test (30-s bins), in males (C) and females (F). α5KO mice showed reduced SD exploration from 210 s onward compared to WT, with α5KI intermediate (genotype × time interaction, RM-ANOVA: males *p* < 0 .0001; females *p* = 0.0016). Abbreviations. M, males; F, females; ND, neutral demonstrator; SD, stress demonstrator. Data are mean ± SEM; n per group indicated in each image.

The recording of the cumulated time spent in the SD zone throughout the session enabled us to visualize the kinetics of the interest exhibited toward the stress demonstrator over the 6-min duration of the trial (Figures 1C and 1F). In WT mice, time spent in the SD zone remained constant throughout the 6-min session, indicating a sustained interest for the SD. However, both male and female α5KO explored the SD zone less from 210 to 360 s compared to WT mice, whereas α5KI exhibited an intermediate performance (genotype effect × time interaction; males: F_(22,231)_ = 2.244, *p* < 0 .0001, partial *η*^2^ = 0.176, 95%CI [0.026, 0.188], females: F_(22,220)_ = 3.118, *p* = 0.0016, partial *η*^2^ = 0.238, 95%CI [0.075, 0.258]). There was no effect of sex on this parameter. Male α5KO mice spent less total time investigating demonstrators compared to WT controls (Figure S3), warranting us to control for differences in social motivation in a separate group of mice. In a three-chamber sociability test (3CST), all males displayed intact social preference (Figure S5A), with all sociability indices (time in social chamber - time in object chamber/time spent in both chambers) significantly greater than zero and no significant group effect (M: F_(2,20)_ = 0.76, *p* = 0.76). In contrast, α5KO females did not show a reduction in the time spent in overall demonstrator investigation compared to sex-matched controls in the ASDT (F_(2,20)_ = 1.46, *p* = 0.26; Figure S3). But, female WT and α5KI groups displayed social preference in the 3CST (Figure S5B), α5KO groups showed a trend that did not reach significance (t(7) = 2.17, *p* = 0.067), and their mean sociability index, while not significantly different from WT, was significantly lower than α5KI mice (F(2,25) = 5.03, *p* = 0.015, partial *η*^2^ = 0.287, 95%CI [0.013, 0.489]; Tukey’s test: α5KO vs. α5KI, *p* = 0.01).

Quantification of sniffing behavior toward SD vs. ND provided further information about affective state discrimination in both nicotinic mutant strains. While WT observer mice showed a clear preference toward SDs, particularly females (t(8) = 9.57, *p* < 0.0001, *d* = 3.19, 95%CI [1.197, 5.183]), this behavior was totally suppressed in most of null mutants (M: t(7) = 0.42, *p* = 0.68; F: t(4) = 0.38, *p* = 0.72; main genotype effect: (F(2,41) = 3.342, *p* = 0.04, partial *η*^2^ = 0.140, 95%CI [0.000, 0.312]) (Figures 2A–2C. For examples of these behaviors see Videos S1 and S2). Counting the number of discriminator (Pref. Score > 10) vs. non-discriminator (Pref. Score < 10) mice within each group revealed further interesting information. First, although both male and female WT mice all performed well on this task, female were unanimous affective discriminators when 22% of male WT were non-discriminators; however, given the small expected cell counts (including a zero cell for female non-discriminators), this comparison did not reach statistical significance (M vs. F: Fisher’s exact test, *p* = 0.47; Figure 2B vs. 2D). The repartition of these two profiles (discriminators vs. non-discriminators) appeared inverted in null mutant male and female mice, although given small expected cell counts these comparisons were assessed using Fisher’s exact test rather than χ^2^. This shift reached significance in females (WT vs. KO: *p* = 0.005) and approached significance in males (WT vs. KO: *p* = 0.057). α5KI mice showed a non-significant, intermediate distribution in both sexes (WT vs. KI: M: *p* = 0.302; F: *p* = 0.206; Figures 2B and 2D). These differences in demonstrator exploration could not be explained by differences in spatial preference or locomotor asymmetry (M: F(2,21) = 0.42, *p* = 0.66; F: F(2,20) = 0.66, *p* = 0.528; Figures S2A and S2B) or by overt displays of anxiety-like behavior as quantified through grooming events during the test (M: F_(2,21)_ = 0.350, *p* = 0.709) although in females α5KO displayed a trend toward lower grooming events compared to the other groups (F: F_(2,20)_ = 3.381, *p* = 0.054; Figure S2C).

**Figure 2:**
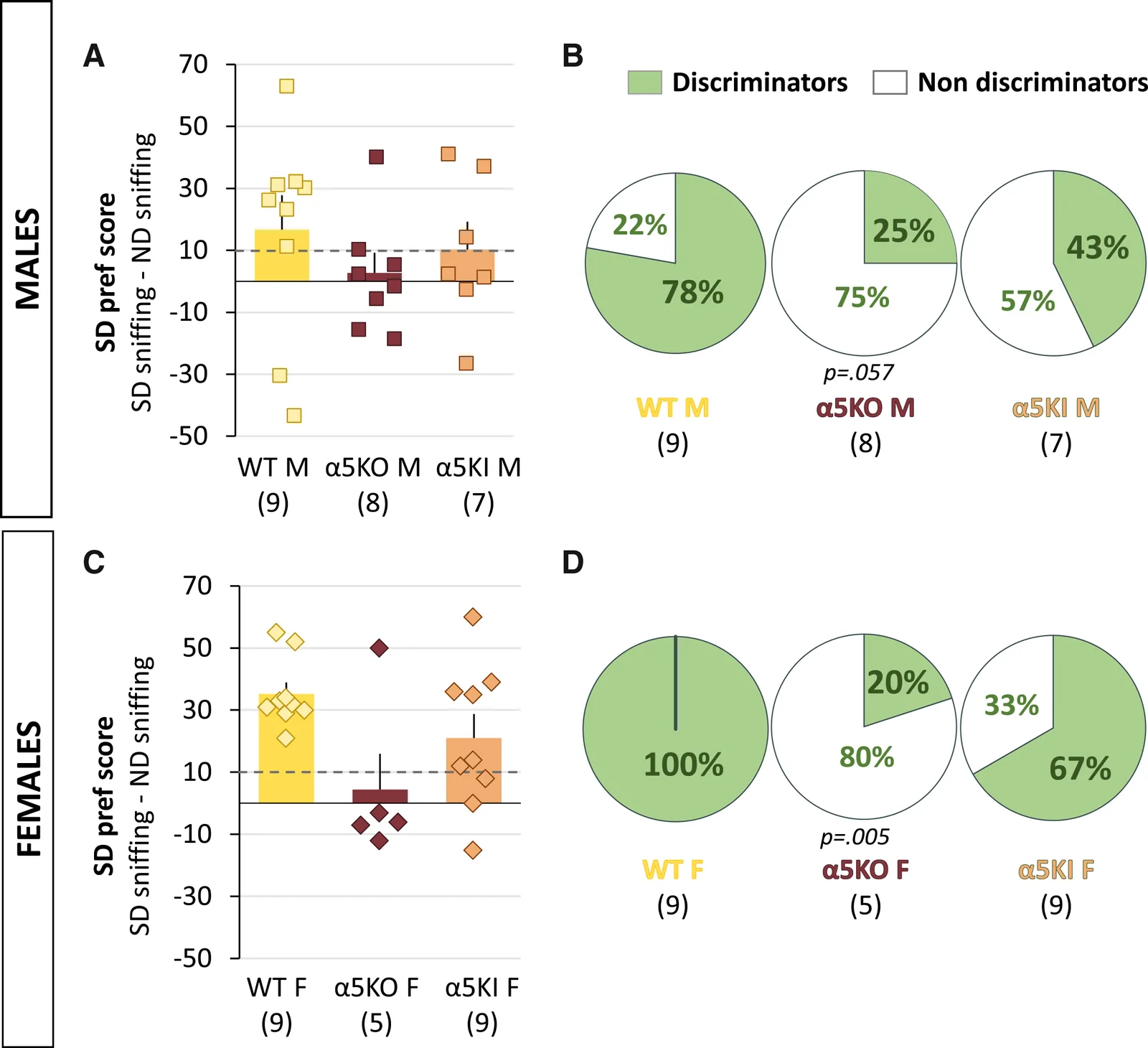
Impairment of exploratory behavior toward a stressed demonstrator in α5 nicotinic mutants (A and C) SD preference score (number of sniffing bouts toward the SD demonstrator minus toward the ND demonstrator) during the test, in males (A) and females (C). A cut-off score of 10 (dashed line) distinguishes discriminators (score > 10) from non-discriminators (score ≤10). WT mice showed a clear SD preference in both sexes, most pronounced in females (paired *t* test: males *p* = 0.17; females *p* < 0 .0001), while this preference was abolished in α5KO mice (males *p* = 0.68; females *p* = 0.72). α5KI mice showed an intermediate profile (males *p* = 0.30; females *p* = 0.026). (B and D) Proportion of discriminators (green) vs. non-discriminators (white) per genotype, in males (B) and females (D). Given small expected cell counts, group comparisons were assessed using Fisher’s exact test. The proportion of discriminators was reduced in α5KO relative to WT, reaching significance in females and approaching significance in males (males *p* = 0.057; females *p* = 0.005), with α5KI showing a non-significant intermediate shift (males *p* = 0.302; females *p* = 0.206). No sex difference was found in WT discrimination rates (Fisher’s exact test, *p* = 0.47). Abbreviations. M, males; F, females; ND, neutral demonstrator; SD, stress demonstrator. Data are mean ± SEM. Each data point represents an individual animal (independent biological replicate).

Video S1. A “discriminator” mouse in the ASDT, related to Figure 2 This recording shows a female WT mouse displaying a preferential approach toward the stressed demonstrator (SD) compared to the neutral demonstrator (ND) during the test trial of the ASDT.

Video S2. A “non-discriminator” mouse in the ASDT, related to Figure 2 This recording shows a male α5KO mouse equally investigating the stressed and neutral demonstrators during the test trial of the ASDT.

### α5KO and α5KI mice display similar cagemate rescue-like behavior

We then aimed to investigate the empathy-related behavior of α5 nicotinic mutants using the Restrainer Tube Task (RTT). Over the first two days, the mice underwent training to learn how to open a paper-sealed tube first from the inside while trapped inside the tube, and from the outside when they were confronted with a tube containing white chocolate chips after being food-deprived for 12 h (Figure 3, top). All mice, regardless of genotype, efficiently learned to open the paper sealed tube from the inside as witnessed by the decrease in their latency to self-freeing (M + F, mean latency on trial 1 vs. trial 6: 335 ± 47.8 vs. 70 ± 9 s); trial effect: F_(5,205)_ = 13.52, *p* < 0 .0001*,* partial *η*^2^ = 0.25, 95%CI [0.138, 0.326]). Notably, females were quicker to free themselves compared to males (mean latency on 6 trials in M vs. F: 91 ± 8.5 vs. 200 ± 21.7 s; sex effect: F_(1,41)_ = 21.26, *p < 0.0001,* partial *η*^2^ = 0.34, 95% CI [0.117, 0.517]*;* trial × sex interaction: F_(5,205)_ = 2.89, *p* = 0.015*,* partial *η*^2^ = 0.07, 95% CI [0.002,0.119]*).* During the three chocolate versus empty trials, all the mice regardless of genotype or sex were quicker to open the chocolate-containing tube on the third trial compared to the first (Figure S4, mean latency on trial 7 vs. trial 9: 1116 ± 97.6 vs. 367 ± 65.6 s; M: F_(1.860, 36.28)_ = 7.091, *p* = 0.003, partial *η*^2^ = 0.267, 95%CI [0.039,0.449]; F: F_(1.820, 36.41)_ = 38.9, *p* < 0 .0001, partial *η*^2^ = 0.66, 95%CI [0.442, 0.758]), indicating that they all efficiently learned how to open a paper-sealed, chocolate-containing tube from the outside, even though the position of the tube was randomized across the three trials. There was no effect of genotype or sex on the opening latency for the chocolate containing tube.

**Figure 3:**
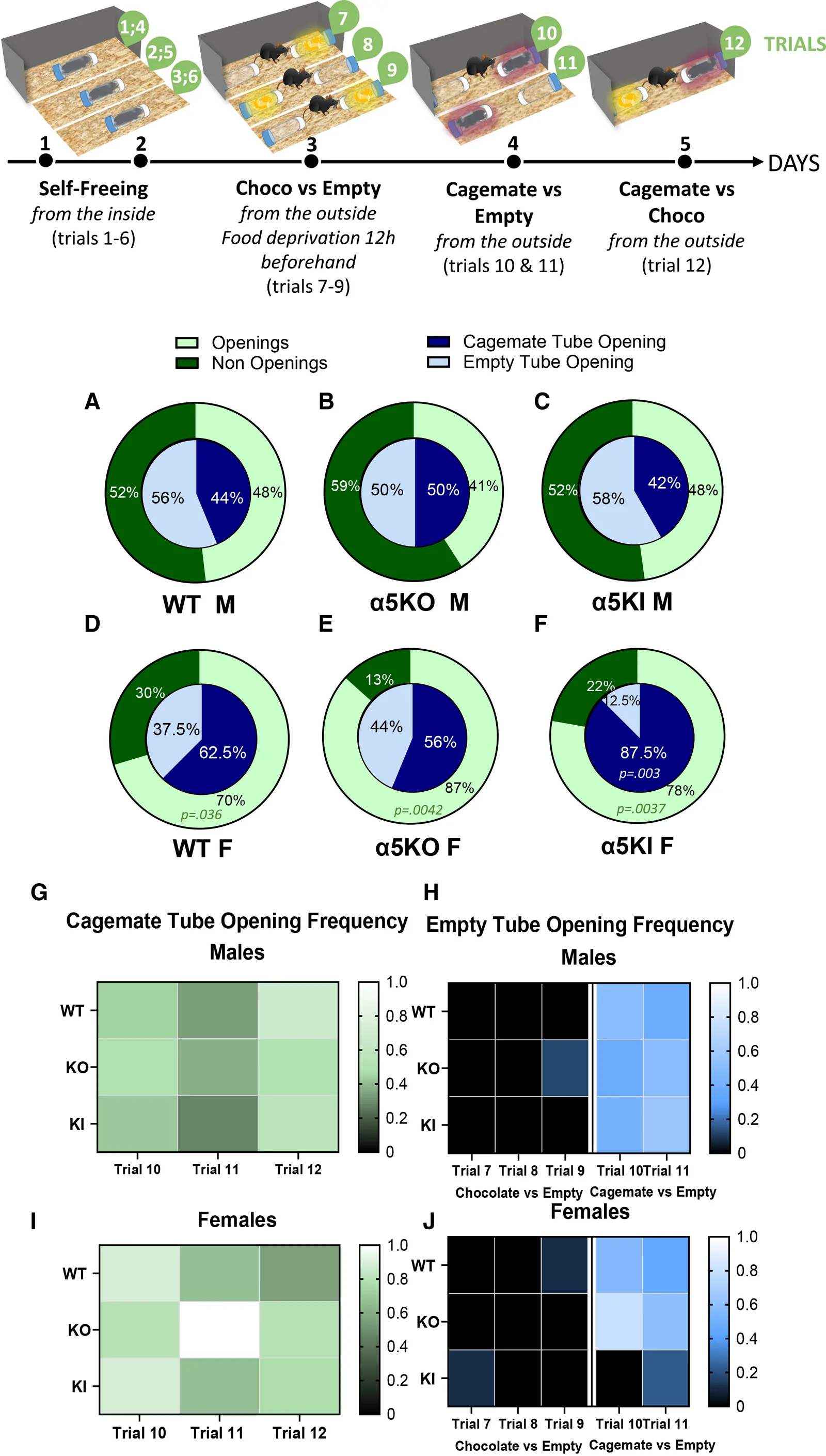
Female α5KI mice exhibit increased cagemate rescuing-like behavior Top: Time course and detailed protocol of the Restrainer Tube Task (RTT) spread over 5 consecutive days (black dots), consisting of a total of 12 trials (green bubbles). (A–F) Pie charts of the proportions of cagemate openings, “Cagemate Openings” were considered as such when the test mouse opened the cagemate-containing tube at least once over the three trials (light green) and “no cagemate openings” considered as such when they failed to open the cagemate tube in any of the three trials (dark green) along with the proportion of cagemate tube openings (dark blue) versus empty tube openings (light blue) in the trials containing a cagemate tube and the trials containing empty tubes respectively for male WT, α5KO and α5KI mice (A, B, and C) and female WT, α5KO and α5KI mice (D, E, F). (G) Frequency chart for cagemate tube openings across the three trials in which this outcome is possible (presence of a trapped cagemate among the presented tubes) in males and in females (I); a lighter gradient corresponds to a higher frequency. (H) Frequency chart for the empty tube opening frequency during the trials in which this outcome is possible; this includes trials in which a chocolate and an empty tube are present in the arena (Trial 7–9) and trials when a cagemate and an empty tube are present in the arena (Trial 10–11) in male and females (J), lighter gradients correspond to higher frequency. CM, cagemate; F, females; M, males. *p* < 0.05, *p* < 0.01: proportion of cagemate-tube openers (outer ring, A-F) significantly different from the 50% chance level (exact binomial test, two-tailed). Significant deviations from equal cagemate/empty tube-opening proportions (inner ring, dark blue vs. light blue) are also indicated where applicable (exact binomial test, two-tailed).

Regarding the cagemate tube opening behavior (Cagemate versus Empty and Cagemate versus Chocolate), a distinction of opening profiles was done based on whether a minimum of one successful cagemate freeing event occurred out of the total three trials (Figure 3, see Video S3; for detailed recurrence of opening over trials, see Figures 4D and 4E). Across all three genotypes, cagemate-tube opening rates in females differed significantly from chance (exact binomial test, two-tailed: WT females, 70.4% opened, *p* = 0.036; α5KO females, 86.7% opened, *p* = 0.0042; α5KI females, 77.8% opened, *p* = 0.0037), while male opening rates did not differ from chance in any genotype (WT: 48.1%, *p* = 0.80; α5KO: 40.9%, *p* = 0.40; α5KI: 47.8%, *p* = 1.0), indicating that females, but not males, reliably freed their trapped cagemate above chance level regardless of genotype. All groups opened both empty and cagemate tubes with a similar frequency (Figures 3G–3J), but females, more specifically α5KI, opened more cagemate tubes than empty ones (respectively: 87.5% versus 12.5%; exact binomial test, *p* = 0.0042; Figures 3I and 3J). This result indicates that the tube opening behavior of α5KI females was guided by the presence of a cagemate inside the tube. Analysis of the trial-by-trial cagemate- and empty-tube opening-profile frequencies (Fisher-Freeman-Halton exact test, given small expected cell counts) confirmed a significant sex difference specifically within the α5KI group (χ^2^(3) = 8.01, exact *p* = 0.025), which was not observed within WT (exact *p* = 0.268) or α5KO (exact *p* = 0.207) mice, indicating a genotype-specific sex effect on cagemate-directed tube-opening behavior.

**Figure 4:**
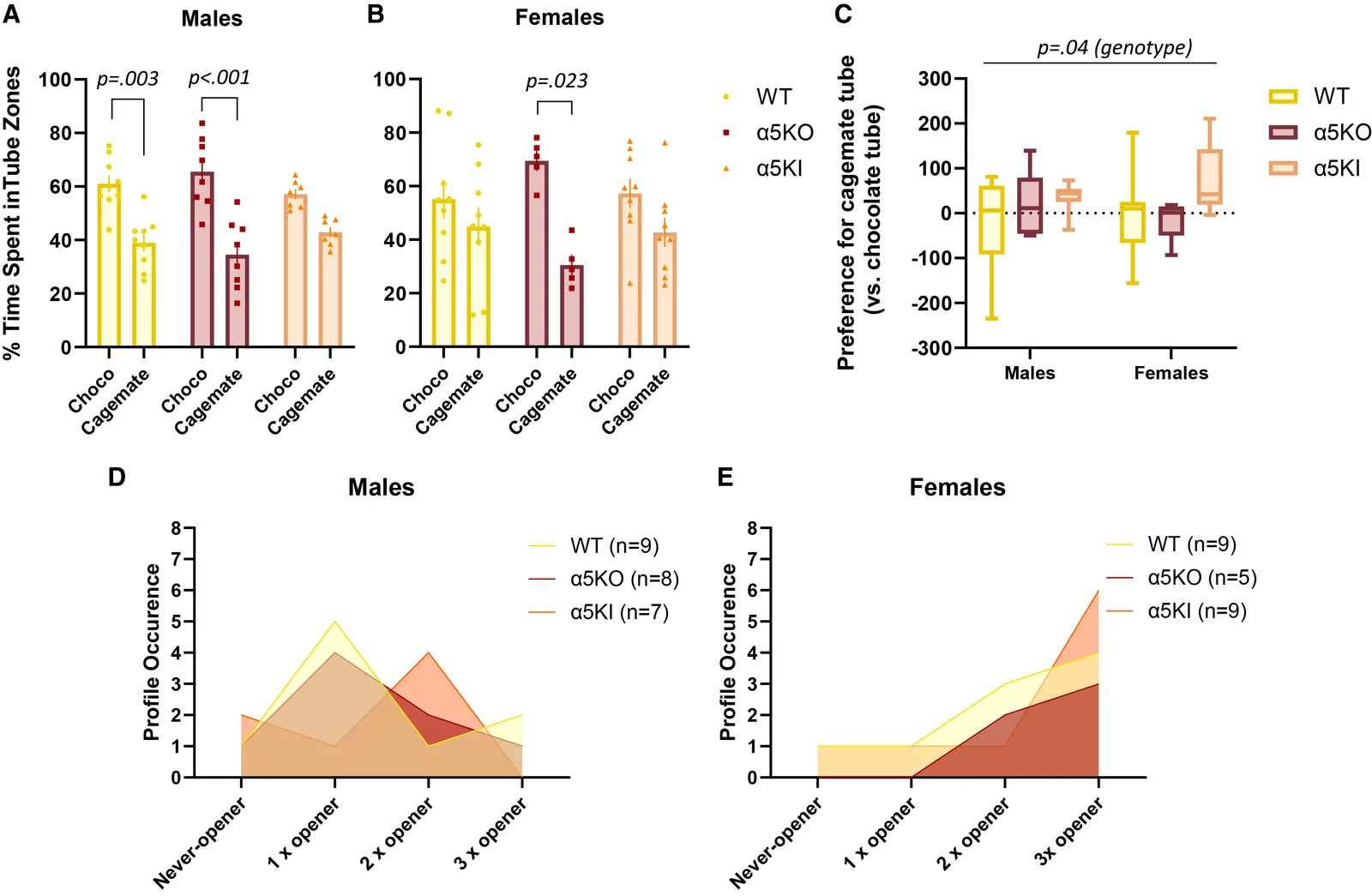
Mice prefer Chocolate over Cagemate containing tubes, but α5KI mice show increased prosocial-like behavior Proportion of time spent in each tube zone during the Cagemate versus Chocolate trial for males (A) and females (B). Cagemate vs. Tube Preference Score corresponding to the number of entries inside the cagemate tube zone subtracted from the number of entries inside the chocolate tube zone across male and female groups of α5KO, α5KI, and WT (C). Exact *p* values are shown for each within-genotype comparison of time spent in the Choco vs. Cagemate tube zone (A, B; paired *t* test, equivalent to repeated-measures ANOVA tube effect); genotype effect on preference score (C): *p* = 0.04 (ANOVA). D, E. Plot of the occurrence of different profiles relating to cagemate opening across each group in males (left) and females (right). (A, B) Data are represented as mean ± SEM, with individual data points shown. Each data point represents an individual animal (independent biological replicate). (C) Boxplots show the median (line), interquartile range (box), and minimum to maximum values (whiskers).

Video S3. A successful cagemate rescuing-like event in the RTT, related to Figure 3 This recording shows a WT female mouse opening the paper-sealed tube restraining its sex-matched cagemate.

### α5KI mice but not α5KO mice display increased empathy-related behaviors

During the competition phase between one tube containing an appetitive reinforcer (i.e., white chocolate chip) and another one containing a cagemate, most of the mice, regardless of their group, spent more time in the surrounding of a chocolate-containing tube when compared to the cagemate tube (tube effect, M + F: F_(1,44)_=27.67, *p* < 0 .0001, partial *η*^2^ = 0.39, 95%CI [0.163, 0.549]; M: F_(1,21)_=29.91, *p* < 0.0001, partial *η*^2^ = 0.59, 95%CI [0.264, 0.735]; F: F_(1,20)_=7.73, *p* = 0.011, partial *η*^2^ = 0.28, 95% CI [0.016, 0.521]; Figures 4A and 4B). However, there was a clear preference of α5KI mice toward the cagemate tube, as compared to both WT and null mutants (expressed as the number of entries inside the cagemate tube zone minus the entries inside the chocolate tube zone). This is supported by a main effect of genotype on this variable (M + F: F_(2,41)_ = 3.42, *p* = 0.042, partial *η*^2^ = 0.14, 95%CI [0.000,1.000]; Bonferroni/Dunn post-hoc, α5KI vs. WT: *p* = 0.011; Figure 4C), indicating an increase in prosocial-like behavior in α5KI mice.

### α5KO and α5KI mice display opposite post-freeing interactions

To further characterize the prosocial profile of both mutant strains, we scored social interactions immediately following the exit of the cagemate from the tube at the end of the test (Figure 5). The results revealed a striking opposition between α5KO and α5KI mice, with genotype effects that differed markedly when analyzed separately by sex. WT mice of both sexes (but more so females) engage mainly in positive interactions after cagemate freeing. In contrast, male α5KO exert higher levels of aggression toward their cagemate as compared to either WT or α5KI males (genotype effect: F_(2,21)_ = 5.27, *p = 0.013,* partial *η*^2^ = 0.33, 95% CI [0.017, 0.538]; Bonferroni/Dunn post-hoc, α5KO vs. WT: *p = 0.0079*, α5KO vs. α5KI: *p = 0.014*; Figure 5A left and Video S4). This aggressive response is not observed in α5KO females, which are predominantly disinterested by the cagemate (genotype effect: F_(2,20)_=3.68, *p = 0.043,* partial *η*^2^ = 0.27, 95% CI [0.000, 0.488]*;* Bonferroni/Dunn post-hoc, α5KI vs. α5KO: *p = 0.014,* α5KO vs. WT: *p = 0.051 ns;* Figure 5A right and Video S5). Conversely, female α5KI express a more interactive, WT-like behavior (genotype effect: F_(2,20)_=3.58, *p = 0.046,* partial *η*^2^ = 0.26, 95% CI [0.000, 0.483]*;* Bonferroni/Dunn post-hoc, α5KI vs. α5KO: *p = 0.014,* α5KI vs. WT: *p = 0.29;* Figure 5A right and Video S6).

**Figure 5:**
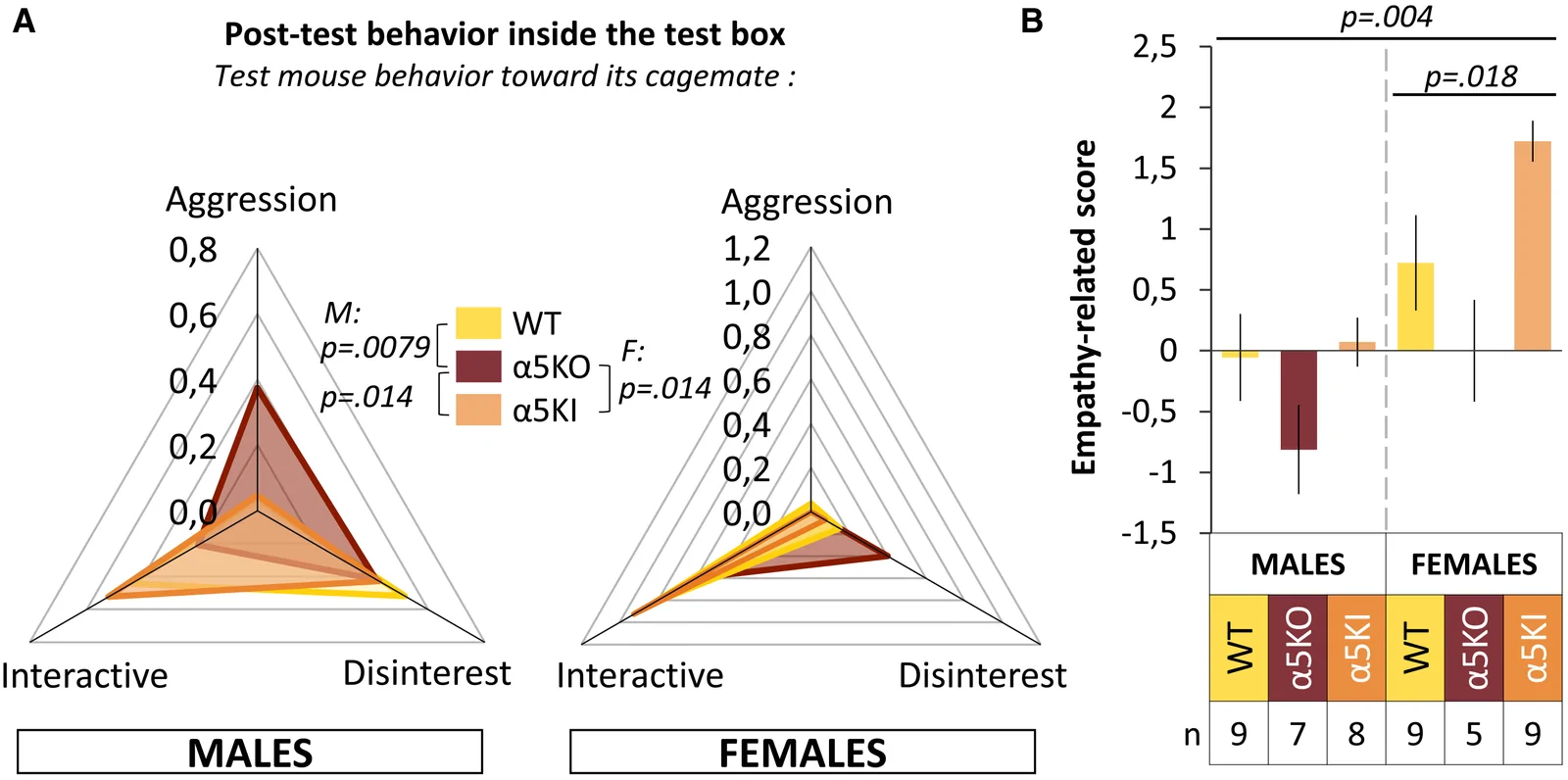
α5 mutants display opposite interaction profiles with the freed cagemate (A) Representation of the frequency of aggressive, interactive, or disinterested interactions for male WT, α5KI, and α5KO mice (left) and female WT, α5KI, and α5KO mice (right). (B) Empathy-related score across male and female nicotinic mutants and controls. The empathy-related score is calculated according to two factors: Cagemate prioritization (depending on which tube is opened first between cagemate, chocolate, and empty) and social-contact positivity according to which type of interaction the mice partake in with the cagemate following the test (Aggression, interactive, or disinterested). *p* < 0.05: Genotype effect (ANOVA); *p* < 0.05: Genotype effect (Kruskal-Wallis test); *p* < 0.05: Genotype effect in females (Kruskal-Wallis test). (B) Data are represented as mean ± SEM.

Video S4. Post-release aggression toward a freed cagemate in an α5KO male mouse in the RTT, related to Figure 5 This recording shows an α5KO male mouse engaging in aggressive behavior toward its sex-matched cagemate following release.

Video S5. Post-release disinterested behavior toward a freed cagemate in an α5KO female mouse in the RTT, related to Figure 5 This recording shows an α5KO female mouse spending minimal time interacting with its sex-matched cagemate following release.

Video S6. Post-release affiliative interaction toward a freed cagemate in an α5KI female mouse in the RTT, related to Figure 5 This recording shows an α5KI female mouse engaging in sustained social investigation of its sex-matched cagemate following release.

Scores of empathy-related behavior based on both cagemate prioritization and prosocial interaction are summarized in Figure 5B. First, males had lower empathy-related scores than females, regardless of the genotype (M vs. F, Mann-Whitney U test: z = −3.33, *p* = 0.0009, r = 0.49, 95% CI [0.237, 0.689]; Figure 5B). Second, α5KO display lower empathy-related scores compared to their sex-matched WT controls (see Video S7 showing an α5KO male mouse opening and exploring the empty tube while ignoring the tube containing its trapped cagemate). Finally, while α5KI male mice show no deficit compared to WT (in contrast to α5KOs), female α5KI mice displayed a higher empathy-related score than any other group, including male α5KI mice and high-performing female WT (Figure 5B). These observations are supported by analyses of scores yielding significant main effect of genotype and sex differences (Kruskal-Wallis test, M + F: H(2) = 11.19, *p* = 0.004, η^2^H = 0.24; M: H(2) = 4.15, p = 0.12 ns; F: H(2) = 7.96, *p* = 0.018, η^2^H = 0.36; Figure 5B). To assess the internal structure of the composite empathy-related score, we examined the correlation between its two components, Cagemate Prioritization (CP) and Social-Contact Positivity (SCP), across the full cohort (*n* = 47, Spearman’s ρ = 0.260, *p* = 0.078). This correlation was significant in females (ρ = 0.440, *p* = 0.036, *n* = 23), particularly in WT females (ρ = 0.815, *p* = 0.007, *n* = 9), but not in males (ρ = −0.183, *p* = 0.391, *n* = 24). In α5KO males specifically, several individuals combined tube-opening (positive CP) with aggressive post-release behavior (negative SCP; ρ = −0.352, *n* = 8), indicating that these two dimensions can be dissociated and supporting the rationale for combining them as complementary components of the composite score. The robustness of the composite score was further confirmed under alternative weighting schemes, including increased weighting of the aggression component and use of cagemate prioritization alone as the sole outcome measure (Table S1).

Video S7. Tube-directed behavior in an α5KO male mouse in the RTT, related to Figure 3 This recording shows an α5KO male mouse opening and exploring the empty tube while ignoring the tube containing its trapped cagemate.

## Discussion

The present study aimed at further characterizing affective state discrimination and rescue-like behaviors in two strains of transgenic mice expressing either a null mutation or a SNP variant of α5∗nAChRs. In humans, GWAS have linked this α5SNP to an increased risk of developing nicotine dependence, schizophrenia, and possibly AUDs. We previously reported that both α5 nicotinic mutants display an increase in chronic, volitional alcohol consumption, while exhibiting an opposite emotional profile reminiscent of the behavioral characteristics of Type I and Type II subtypes of patients with AUD.^7^ Considering the transnosographic importance of social abilities, we aimed to further investigate the social-emotional profile of α5 nicotinic mutants. The present study also contributes to the characterization of sex differences in these behaviors.

We first showed that, in a task aimed at distinguishing a stressed unfamiliar conspecific from a neutral one (ASDT), female WT mice were better emotional discriminators than males, exhibiting a preferential approach toward the stressed demonstrator twice superior to the males, along with a markedly stronger sniffing preference for the SD over the ND demonstrator. Descriptively, females were also unanimous discriminators using our categorical cut-off, while 22% of males were not, although this categorical difference did not reach statistical significance. These results are in line with previous studies suggesting that female mice may have a better ability to detect negative emotions in conspecifics.^55^^,^^56^ In contrast to sex-matched WT, however, neither male nor female α5KO mice did preferentially approach a stressed unfamiliar conspecific compared to a neutral one, thus revealing a deficit in their ability to perceive negative states in peers. As compared to null mutants, α5KI mice carrying the α5SNP were less impacted in the ASDT, although they were less efficient than sex-matched WT. Importantly, female α5KI also displayed higher ASDT performances than α5KI males.

Affective state discrimination in mice has been shown to rely on activation of somatostatin-expressing (SOM) interneurons of the PFC^43^ and on oxytocinergic projections from the paraventricular nucleus of the hypothalamus to the amygdala.^39^^,^^43^ α5-nAChRs are expressed at key nodes within these circuits: in vasoactive intestinal peptide-expressing (VIP) interneurons that exert inhibitory control on SOM interneurons in the PFC,^50^ and in oxytocin-containing magnocellular neurons of the hypothalamic paraventricular nucleus.^57^ Moreover, blocking oxytocin receptors in the PFC of rats interferes with the pro-social motivation to free a trapped conspecific.^58^ The functional properties of the α5 subunit are therefore mechanistically relevant: although accessory and not required for receptor assembly, α5 enhances receptor efficacy and calcium permeability when incorporated into the pentamer. The *Chrna5* SNP *rs16969968* carried by α5KI mice causes a partial loss of function of α5∗nAChRs, characterized by reduced intracellular Ca^2+^ responses and decreased receptor efficacy.^59^^,^^60^ The genetic knockout of α5, by contrast, results in the complete absence of the subunit, with the vacated position within the pentamer occupied by an alternative subunit and a consequent alteration in receptor stoichiometry. The behavioral changes observed in α5KO and α5KI mice may therefore reflect differential dysregulation of α5∗nAChR-mediated control over PFC SOM interneurons and hypothalamic oxytocinergic neurons, a neuromodulatory system central to empathy-related and prosocial behavior.^27^^,^^61^ The ventral hippocampus represents an additional candidate substrate, given its established role in affective empathy as assessed by observational fear paradigms in rodents.^62^^,^^63^

Regarding the prosocial-like act of rescuing conspecifics from the situation of spatial constraint (RTT), we first observed that WT females freed a sex-matched cagemate from constriction tubes while most of males did not. Males were more interested in alternative tubes (empty or containing white chocolate) than ones containing a trapped cagemate, and were less interested in interacting with the freed cagemate during the post-test phase, or did so antagonistically. This is consistent with previous reports showing that female rats are more motivated to free their trapped cagemates.^45^ Indeed, it has been suggested that in many species, including humans, females show more evidence of empathy-driven behaviors than males cagemate.^64^^,^^65^^,^^66^ However, in another study up to 70% of male mice were found to open the tubes restraining their sex-matched cagemate^45^^,^^48^ and, in contrast to the present data, subjects did not open alternative empty tubes. One explanation for these differences could be the mouse strain, as we used C57BL6/J mice and not C57BL6/N as in Ueno et al.,.^48^ A recent study demonstrated that mice from these two substrains exhibit behavioral and physiological differences,^67^ that may also include empathy-related behaviors underlying this task. Another explanation could be the age of male subjects, as ours were 24 weeks old (mature adults) instead of 10 weeks old (young adults). In humans, older adults tend to exhibit more emotional empathy and pro-social behavior than younger adults.^68^^,^^69^ However, age-related effects on empathy-related behaviors have not been investigated in rodents yet. Whether empathy-related and prosocial-like responding in mice follow an age-dependent trajectory analogous to that reported in humans remains untested; repeating the ASDT and RTT in wild-type mice across several life stages (e.g., young adult, mature adult, aged) would help establish whether an aged-response curve exists in mice and would further strengthen the translational validity of these paradigms as models of empathy-related behavior. Also, we did not observe any chocolate sharing-like behavior in both male and female WT mice; but as Bartal et al.^45^ found that rats shared chocolate chips with their freed cagemate in only half of all trials, this is likely due to the fact that we performed only one chocolate vs. cagemate trial. More trials would be needed to verify whether mice also expressed chocolate sharing-like behavior.

In the RTT, unlike WT mice, α5KO mice showed a reduced prosocial-like response, which could be interpreted as a decrease in motivation to help their distressed peers. Similar to sex-matched controls, the majority of α5KO males did not open the tube restraining their cagemates and primarily opened the alternative tubes, including chocolate-containing and empty ones. However, when it came to the post-test social interaction, α5KO males differed from sex-matched controls by exerting high levels of aggression, which in humans is associated with deficient affective empathy.^32^ In light of recent studies showing aggression self-administration in male mice,^70^ it is possible that the opening behavior of some α5KO males was motivated by aggression seeking. Such an increase in aggression was not observed in α5KO females, who rather showed an increase in cagemate freeing. But in contrast to sex-matched controls, α5KO females spent more time around the chocolate-containing tube than the cagemate-containing one and did not display interactive behavior toward the freed cagemate. The latter parameters, coupled with their inability to discriminate negative from neutral emotional states in conspecifics, suggest that α5KO females were more likely to free their cagemate because they enjoy the task of tube opening^48^ rather than because they were motivated by rescuing the distressed cagemate. Notably, α5KO mice displayed a modest reduction in social interest at baseline: males explored demonstrators less during the ASDT, while females showed reduced social preference, raising the question of whether impaired affective discrimination could reflect generalized social withdrawal rather than a specific deficit in emotional processing. Two lines of evidence argue against this interpretation. First, both demonstrators were presented simultaneously, meaning any generalized reduction in social interest would be expected to reduce exploration of both equally. Consistent with this, normalizing the SD preference score to total demonstrator investigation time did not alter the pattern of results (Figure S1). Second, and more decisively, the RTT data are difficult to reconcile with simple social disinterest: α5KO mice retained the capacity to free a restrained cagemate yet consistently engaged in antagonistic interactions upon doing so. A mouse indifferent to social stimuli would not be expected to open a cagemate’s tube and then attack it. This pattern is more consistent with an active disruption of prosocial-like behavior: closer to an “anti-social” profile than a withdrawal one. Taken together, alongside previously published results of low anxiety, impulsivity and high voluntary alcohol consumption, these findings fit the characteristics of Type II AUD.^7^^,^^14^^,^^16^^,^^31^

In addition, female α5KI mice stood apart from both WT and α5KO females by displaying markedly more interactive behavior toward the freed cagemate and by overwhelmingly opening more cagemate-containing tubes than empty ones, indicating that their rescuing-like behavior was socially motivated rather than driven by the act of tube-opening itself. This distinction is sharpened by the design of the paradigm, inspired by Bartal et al.,^45^ which contrasts the social stimulus against a competing appetitive reinforcer (chocolate) as a stringent test of social salience. Whereas all groups dwelled longer near the chocolate tube than the cagemate tube, female α5KI nonetheless entered the cagemate zone more frequently. For this group, cagemate approach survived competition with the primary reinforcer. The convergence of cagemate tube preference over empty, resistance to chocolate competition, and positive post-release social interaction delineates a profile qualitatively distinct from both WT and α5KO mice, and one strongly evocative of cagemate prioritization and consolation-related behavior.

It is worth noting that the major difference between the two social procedures was the affiliation between the subject and the non-subject mice: in the RTT, the distressed mouse to rescue” was a littermate, while the demonstrators to discriminate in the ASDT were unfamiliar mice. A previous study showed that male α5KI mice display a reduction in approach to a novel unfamiliar mouse compared to controls in a three-chamber sociability test.^50^ Moreover, our lab demonstrated that α5KI mice are hyper-anxious.^7^ It was previously reported that, in mice and human subjects, empathy for strangers is blunted by social stress, and blocking glucocorticoid synthesis or glucocorticoid or mineralocorticoid receptors enhances empathy in the same situation.^71^ It is therefore possible that the hyper-anxious and novelty-avoidant phenotype of α5KI mice makes them perceive the stressed unfamiliar conspecific as a threat, which could hamper their exploration and explain why they were found to be bad affective state discriminators in the ASDT. The score for empathy-related behavior, which takes cagemate prioritization and social-contact positivity into account during the RTT, enabled us to get a good overview of the interaction of how sex and genotype jointly shape the display of pro-social behavior across groups. While null mutants scored negatively in both sexes, female α5KI scored positively compared to controls making them the group with the most display of responsiveness to a conspecific’s distress and strong cagemate-directed behavior, consistent with an empathy-related interpretation. The behavior of α5KI females particularly evoked the affiliatively motivated dimension of type I AUD profile,^14^ which, together with prior work showing their elevated anxiety and alcohol consumption,^7^ further supports their resemblance to the features of this particular subtype.

It should be noted that male and female mice differ intrinsically in how they respond to unfamiliar conspecifics, with males typically displaying higher levels of territorial aggression toward novel males and females showing comparatively greater social tolerance and affiliative behavior.^72^^,^^73^ This baseline difference is most relevant to the ASDT, which involved unfamiliar demonstrators, and may have contributed to the lower and less unanimous approach observed in WT males relative to females. It is less directly applicable to the RTT, since the trapped/free mice were familiar cagemates rather than novel conspecifics; nonetheless, established dominance relationships between cagemates could plausibly have shaped both the decision to open the tube and the nature of the post-release interaction. Rodents living in social groups, including mice, spontaneously form dominance hierarchies that are relatively stable over time.^72^^,^^74^ While this phenomenon has historically been characterized mainly in males, recent work indicates that female mice also establish hierarchies, albeit typically less despotic ones, accompanied by rank-related physiological differences such as basal corticosterone levels.^75^^,^^76^^,^^77^ Social rank is known to influence anxiety-like behavior, stress reactivity, and aggression,^78^^,^^79^ all of which could plausibly modulate performance in both the ASDT and RTT. In our study, the dominance status of cagemates prior to testing was not assessed, and we cannot rule out that pre-existing dominant/subordinate relationships influenced whether a subject mouse approached, opened the tube for, or interacted aggressively with its cagemate, particularly for α5KO males, whose post-release aggression could conceivably reflect a rank-related dynamic rather than (or in addition to) a genotype-driven empathy-related deficit. Future studies should incorporate an independent assessment of social dominance (e.g., tube test) prior to behavioral testing, so that rank can be considered as a covariate in interpreting sex- and genotype-dependent prosocial-like and empathy-related responses.

The empathic motivation to perform rescuing-like behavior in laboratory rodents is still widely debated.^34^^,^^42^^,^^48^^,^^80^^,^^81^^,^^82^^,^^83^^,^^84^^,^^85^^,^^86^ The current work supports that the opening behavior must be carefully interpreted. Relying only on the data put forth, we cannot formally distinguish between prosocial motivation, heightened social attachment, anxiety about cagemate distress, or motivation to terminate distress signals as a cause for the cagemate freeing in the RTT. But these are not mutually exclusive with an empathy-related interpretation: anxiety about a cagemate’s distress and motivation to terminate it are themselves components of affective empathy-related rather than alternatives to it. We suggest that a social release paradigm such as the RTT must be coupled with at least one other task assessing the ability to recognize a distress state, which is a prerequisite to express pro-social helping behavior. Analyzing the post-test interaction between the subject mouse and the trapped mouse is also essential because an opening behavior coupled with aggression or complete disinterest toward the distressed cagemate, as observed in α5KO, cannot be categorized as empathic-like or pro-social. However, an opening behavior followed by a positive interaction with the freed cagemate may either support the seeking of social contact as a rescuing motivation^87^^,^^88^ or reveal an interest toward a distressed cagemate (and maybe some premises of consolation-like behavior). Thus, studying the relative interest for alternative tubes is also required. Prioritization of cagemate tube opening over alternative tubes as observed in α5KI females may reflect either the desire to “rescue” a peer or the desire to stop their stress signals.^89^ However, neither the act of opening alternative tubes, nor the act of entering it can deny the existence of empathic-like concerns. Indeed, the subject mice first learned to open trap tubes by being themselves restrained in them. During the next trials, they later had to understand that cagemates trapped inside the same device could not free themselves. Thus, the act of opening and entering alternative tubes may reflect imitative behavior, while waiting for the cagemate to free itself, rather than a higher interest in the restraint tool as proposed by Ueno, 2019. In support, these behaviors were mostly observed in cagemate vs. empty and not in chocolate vs. empty trials. We point out that establishing an empathy-related score with several variables collected in the social release paradigm (opening behavior, cagemate prioritization over alternative tubes, and positivity of post-test interaction) provides many analogies with human data, such as sex-stratified profiles and the genotype-dependent changes in nicotinic mutant strains. The translational analogies advanced here would be strengthened by within-animal correlations between empathy-related measures and alcohol phenotypes, by testing whether alcohol exposure modifies ASDT or RTT performance across genotypes, and by replicating the social-emotional profiles across additional paradigms such as social memory, observational fear, and consolation-like behavior. Overall, we demonstrate the critical role of α5 nicotinic receptors in affective state discrimination and rescue-like behaviors in response to congeneric stress. We also reveal important effects of sex in WT animals, with genotype effects that were frequently sex-stratified across the behavioral endpoints examined. The results of our study further suggest that genetic mutations targeting α5 receptors could be a common cause of the socio-emotional deficits observed in various pathological conditions, such as addictive behaviors and schizophrenia. Future research should investigate the functional consequences of α5∗nAChR mutations at the neural network level.

### Limitations of the study

Several limitations should be acknowledged. No physiological readouts of emotional contagion were collected; quantifying plasma corticosterone immediately after the ASDT and RTT, recording ultrasonic vocalizations (USVs), and monitoring freezing as an index of arousal and affective reactivity during the tasks would provide valuable complementary indices, given that corticosterone rises during emotional discrimination in rodents^90^^,^^91^ and that emotional states are actively communicated through USVs. Additionally, estrous cycle monitoring was not conducted in the present cohort; given documented influences of cycle stage on socio-emotional behavior and stress reactivity,^92^^,^^93^^,^^94^ systematic cycle tracking should be prioritized in future work to clarify the hormonal modulation of the female phenotypes reported here. Future work should also include conditions involving a familiar non-cagemate and an unfamiliar conspecific in the RTT, which would help determine whether the post-release aggression observed in α5KO males is specific to the context of liberating a distressed familiar cagemate, or reflects a more generalized agonistic social style. Although WT mice originating from the α5KO and α5KI lines did not differ significantly on the ASDT and RTT parameters tested (see Methods), we cannot fully exclude subtler effects of line-specific cage environment or maternal factors on WT behavior beyond the measures assessed here. We note, however, that using littermate WT controls from each line, rather than an independent, non-littermate WT cohort, has the advantage of matching maternal environment, early-life cage conditions, and genetic background between each mutant strain and its own WT controls, which helps disentangle genotype-driven effects from strain-of-origin or husbandry confounds. Future studies could further strengthen this by tracking cage-of-origin as an explicit covariate in larger cohorts. Furthermore, we cannot entirely exclude that baseline differences in anxiety, stress reactivity, or attentional processing contribute to the social-emotional phenotypes observed.^7^^,^^50^^,^^95^ A more complete within-cohort behavioral characterization, including concurrent measures of anxiety, impulsivity, and novelty avoidance in the same animals, would further strengthen these conclusions. The present interpretation instead relies on integrating the current findings with our prior characterization of these lines,^7^ a common approach in behavioral genetics but one that cannot fully rule out a contribution of individual variation in baseline emotional reactivity to the phenotypes reported here. However, locomotor activity was comparable across genotypes, both strains displayed intact sociability, and performance measures were normalized to control for individual differences in total investigation time. The convergence of findings across two independent paradigms further supports a genuine and opposing divergence in social-emotional processing in α5KO and α5KI mice, rather than a generalized sensory or motivational confound. Finally, the α5KO female subgroup was limited to *n* = 5, constraining statistical power and generalizability. The consistently large effect sizes across endpoints suggest the patterns are not spurious, but replication in larger cohorts is necessary before firm conclusions can be drawn for this specific subgroup.

## Resource availability

### Lead contact

Requests for further information and resources should be directed to and will be fulfilled by the lead contact, Léa Tochon (ltochon@rockefeller.edu).

### Materials availability

This study did not generate new unique reagents.

### Data and code availability

• The data supporting the findings of this study, including animal-level and trial-level source-data files for all behavioral endpoints and representative videos, are available within the article and its supplementary information files (Data S1, S2 and S3), and have been deposited in Mendeley Data: https://data.mendeley.com/datasets/xssw5fd2mh/2.^96^ Any additional data not included in these files are available from the lead contact upon request.
• This paper does not report original code.
• Any additional information required to reanalyze the data reported in this paper is available from the lead contact upon request.

## Acknowledgments

We thank Uwe Maskos (Institut Pasteur, Paris) for providing us with the initial mouse founders for the local breeding of α5KO and α5KI (α5SNP) mice. This work was supported by the 10.13039/501100004794Centre National de la Recherche Scientifique CNRS UMR 5287 and Bordeaux University, 10.13039/501100001665Agence Nationale de la Recherche (ANR), French National Cancer Institute (INCa/IResP/SPA 2021-002 and INCa/2024-198 grants). The laboratory of VD was part of the Bordeaux Neurocampus and a member of the Laboratory of Excellence network (LabEx BRAIN), and now of the Great Research Project (GPR) Plan. As such this work was supported by French state funds managed by the 10.13039/501100001665Agence Nationale de la Recherche (ANR) within the Investissements d’Avenir program under reference ANR-11IDEX- 0004-02.

## Author contributions

Conceptualization, V.D. and L.T.; methodology, V.D., N.H., C.P., and L.T.; data analysis, C.P. and L.T.; writing – original draft preparation, C.P. and L.T.; writing – review and editing, all authors; supervision, V.D. and J.L.G.; project administration, V.D.; funding acquisition, V.D. All authors have read and agreed to the published version of the manuscript.

## Declaration of interests

The authors declare no competing interests.

## Declaration of generative AI and AI-assisted technologies in the manuscript preparation process

During the preparation of this work, the authors used Claude (Anthropic) to assist with rephrasing manuscript text. The authors reviewed and edited all AI-assisted content and take full responsibility for the integrity and accuracy of the published work.

## STAR★Methods

### Key resources table

**Table:** 

REAGENT or RESOURCE	SOURCE	IDENTIFIER
**Deposited data**		

Animal-level and trial-level source data for all behavioral endpoints (ASDT, RTT, three-chamber sociability test)	This paper	Mendeley Data: https://doi.org/10.17632/xssw5fd2mh.2

**Experimental models: Organisms/strains**		

Mouse: α5KO (Chrna5 knockout, C57BL/6J background)	Salas et al., 2003; provided by Dr. Maskos (Institut Pasteur) and maintained for 8 additional generations in our laboratory	https://doi.org/10.1124/mol.63.5.1059
Mouse: α5KI (α5SNP rs16969968 knock-in, C57BL/6J background)	Koukouli et al., 2017; provided by Dr. Maskos (Institut Pasteur) and maintained for 8 additional generations in our laboratory	https://doi.org/10.1038/nm.4274

**Software and algorithms**		

GraphPad Prism	GraphPad Software	RRID:SCR_002798
StatView	SAS Institute Inc.	RRID:SCR_017411
ANY-maze	Stoelting Co.	RRID:SCR_014289

### Experimental model and study participant details

#### Animals and housing

We used two transgenic mouse strains expressing different mutations of the *Chrna5* gene encoding for the α5 subunit of nAChRs, on a C57BL/6J background. The α5KO mice carry a targeted deletion of *Chrna5*, originally generated using 129/Sv embryonic stem cells and subsequently backcrossed onto a C57BL/6J background; these mice were first produced by Dr. Beaudet’s group.^97^ The α5SNP mice (referred to as α5KI hereafter; *rs16969968* knock-in) express a single nucleotide polymorphic variant defined as a substitution of aspartate by asparagine (D398N in humans, D397N in mice), corresponding to the most frequent α5-SNP found in the Western population^8^; these mice were derived from the line generated by Dr. Stitzel’s group^50^ generated by homologous recombination in C57BL/6 embryonic stem cells using BAC-derived constructs (Ingenious Targeting Laboratory), with the neomycin selection cassette excised by crossing with C57BL/6J Zp3-Cre mice (Jackson Laboratory), with successful excision confirmed by PCR. Both lines were initially provided by our collaborators Drs. Maskos and Besson at the Pasteur Institute (Paris) and were bred in the animal facility of the lab for 8 generations, with a cross-breeding with heterozygous mice from the Pasteur Institute at the 4th generation. Wild-type mice were obtained from the same litter as both α5 nicotinic mutants. Genotype identifications were conducted at 7 post-natal days (P7) through PCR analysis of tail samples by the genotyping platform of the Neurocentre Magendie in Bordeaux. Each line (α5KO, α5SNP/α5KI) was bred from heterozygous crosses, so litters and home cages typically contained a mix of WT, heterozygous, and homozygous mutant littermates. Mice were housed by line of origin, with WT and mutant littermates from the same line co-housed together. For the experiments, we used mice aged of 20–24 weeks. The mice were housed in grouped cages (4–8 per cage) with access to water and food *ad libitum* in a temperature-controlled room (22 ± 1°C). The lighting of the cages followed a 12-h day/night cycle in such a way to allow testing to occur from 7:00 a.m. to 07:00 p.m. during the mice’s light-on cycle. All experimental procedures were approved by the Ethics Committee of Bordeaux University (CEEA-050) and the French Ministry of Higher Education and Research, under authorization number APAFIS#12283 (approved 06/25/2018) and APAFIS #35136 (approved 17/06/2024), in agreement with Directive 2010/63/EU.

#### Group repartition

Each mouse underwent two behavioral tasks: the affective state discrimination task (ASDT), and the restrainer tube test (RTT) 4 weeks later. Order was not counterbalanced, as the RTT’s food deprivation and tube-training requirements made it preferable to run second, after the non-food-restricted ASDT. Both male and female mutants (α5KO: *n* = 8 ♂ and 5 ♀; α5KI: *n* = 7 ♂ and 9 ♀) as well as male and female WT littermate mice (*n* = 9 ♂ and 9 ♀) were included in these experiments. Wild-type (WT) mice were littermates of both the α5KO and α5KI lines and were pooled into a single WT control group per sex, rather than testing a separate task-specific WT cohort for each line: both the ASDT and RTT are established paradigms from the literature rather than novel assays being validated, and pooling littermate WT controls from both lines avoided introducing a substantial sample-size imbalance relative to each mutant group. Among the 9 male WT mice, 4 were α5KO littermates and 5 were α5KI littermates; among the 9 female WT mice, 4 were α5KO littermates and 5 were α5KI littermates. To assess whether line of origin influenced WT behavior, WT mice from the two lines were compared separately within each sex on key ASDT parameters (time spent with the stressed vs. neutral demonstrator, SD-ND preference score, sniffing duration, total distance traveled) and RTT parameters (tube entries and dwell time near the chocolate-vs. cagemate-containing tube) using Mann-Whitney U tests. No significant differences were found between WT subgroups of different line origin on any parameter, in either sex (all *p* > 0.11), supporting their pooling into a single WT control group per sex. No animals were excluded from either task.

### Method details

#### The affective state discrimination task (ASDT)

We used an adaptation of the ASDT protocol described by Scheggia and colleagues,^43^ which consist in determining the preferential approach tendency in observer mice when exposed to two unfamiliar demonstrator mice in different affective states.^39^^,^^43^ This aimed at testing the capability of mice to discriminate between a neutral demonstrator and a stressed demonstrator, as part of an affective state discrimination task. Briefly, the observer was introduced in a cage (29 × 35 × 19 cm) containing two demonstrator mice placed in transparent plastic cylinders (H:21 cm; D:10 cm) with perforated holes at the bottom to allow for breathing and sensory interaction with the environment. A metal separator (2 mm × 21 cm x 18 cm) was placed in the middle of the cage in order to mark off the two areas of interest corresponding to each demonstrator and blocking their reciprocal view of each other. Demonstrators were WT mice and were sex-matched to the observer. The entire testing arena was wiped down with warm water and the litter was changed between each subject to eliminate any odors left by the mice. At the end of each testing day, the entire apparatus was wiped down with Ethanol (70%).

#### Habituation

All the mice (demonstrators and observers) were handled for 5 min a day over 3 days to become familiar with the experimenter.^98^ They were then habituated to the testing zone for 10 min a day over another 3 days. For the test zone habituation, observer mice were gently placed in the ASDT arena and allowed to freely explore in the absence of the demonstrator mice inside each cylinder (Figure 1, top). During the habituation, the mice’s movements were tracked with a tracking Software (Anymaze, Stoelting). After the observer mouse’s habituation trial ended, its allocated pair of demonstrator mice was subsequently gently placed inside their corresponding cylinder for 10 min.

#### Testing

For the purpose of creating different affective states in mice, the demonstrators were divided into 2 groups: Neutral and Stress. Stress demonstrators (SD) underwent a restraint procedure for 15 min prior to the beginning of the test which consisted in constraining them inside a 50 mL custom-made tube (Falcon©) perforated at the end to allow for breathing. After 15 min of restraint, the stressed demonstrator was immediately placed in one of the two cylinders which was marked by a red-lid for identification. Neutral demonstrators (ND) were not further handled in a way to avoid altering their emotional state and were directly transferred from their home cage to the other cylinder which was marked with a blue lid. For each test trial, ND and SD mice were randomly allocated either to the right or left cylinder relative to the central separator, during both habituation and testing trials, to avoid the confounding variable of spatial preference. The observer was then gently introduced inside the arena and its exploration was recorded for 10 min using Anymaze. Demonstrator zones (SD and ND zones) were outlined using the tracking software and consisted in a square perimeter of 2 cm around and including the cylinders. Parameters of interest such as: time spent and number of entries inside the zone were automatically recorded. In addition, sniffing of the demonstrators were scored a posteriori blindly using the offline recordings.

#### ASDT parameters

Parameters recorded were time spent around each demonstrator zone (SD and ND zones). Quantification of sniffing behavior toward SD vs. ND was used to establish a preference score defined as SD sniffing – ND sniffing. A sniffing event was operationalized by the occurrence of a nose contact of the observer toward the cylinder containing a demonstrator. We then categorized each group into discriminators by using a cut-off score of 10 sniffings (Pref. Score >10; high discriminators) versus non-discriminators (Pref. Score <10).

#### The restrainer tube test (RTT)

The Restrainer Tube Test (RTT) aimed at exploring pro-social rescuing-like behavior through the testing of social release. We based our testing procedure on the ones used by Bartal et al.^45^ and Fontes-Dutra et al.^40^ in their studies measuring the willingness of rats to free their cagemate trapped in a tube and its adaptation to mice by Ueno et al.^48^ The test was carried out over 5 days following 4 different phases shown in Figure 3: Self-freeing training (Day 1 & 2), Chocolate versus Empty tube (Day 3), Cagemate versus Empty tube (Day 4) and a competition trial: Cagemate versus Chocolate tube (Day 5). In contrast to protocols established for rats in which trap devices may not be stressful enough,^99^ the adaptation to mice using a restraint tube ensure a significant distress.^100^ Moreover, we develop a short procedure with only 2 days of exposure to entrapment (for both subject and cagemate mice), this period of time is not sufficient for the mice to develop stress habituation.^101^

#### Self-freeing training

The mouse was placed in the restraint tube (50 mL plastic tube, Falcon©) closed off with a paper door at the extremity facing the head of the animal inside the testing cage, the mouse therefore learned to free itself from the inside of the tube by chewing through the paper door. The training was done three times a day over two days (3 trials on Day 1 + 3 trials on Day 2; Figure 3, top). All the mice successfully learned how to open the tube from the inside after 2 days of training.

#### Chocolate opening training

The mouse was trained to open the paper door on a restraint tube from the outside to access white chocolate chips (3 × 25 mg chips). In order to motivate the animals for the task, they were food-deprived during the 12 h preceding the training. The mouse was placed inside the testing cage with an empty and a chocolate containing tube closed off with a paper lid and was left to roam freely until it had opened the chocolate tube and eaten the chocolate chips. This training was done 3 times over one day (3 trials on Day 3; Figure 3).

#### Cagemate freeing training

Each test mouse was assigned a sex-matched ‘trapped cagemate’ consisting in a heterozygous (±) littermate that was trapped inside a paper sealed tube at its rear so that the trapped mouse could not free itself. The test mouse was placed in the cage with the cagemate-containing tube as well as an empty paper-sealed tube. In order to free its cagemate, the test mouse needed to chew through the paper door from the outside. The session ended 5 min after the mouse had successfully freed its cagemate, or after a maximum of 90 min had elapsed. The training was repeated twice over one day (2 trials on Day 4; Figure 3).

#### Chocolate versus cagemate trial

The final trial of the test consisted in placing the mouse in the testing arena in the presence of a chocolate-containing tube (3 × 25 mg white chocolate chips) and a cagemate-containing tube, both closed off with a paper lid (1 trial on Day 5; Figure 3). For all trials, the latency to open the tubes as well as the order of opening of the tubes was recorded. Additionally, the number of white chocolate chips eaten by the test mouse was counted to account for sharing-like behavior. A digital camera was placed above the testing apparatus to record the Chocolate versus Cagemate Trial with a behavioral tracking system (Anymaze, Stoelting). Tube zones were outlined with the software which consisted in a rectangular perimeter of 1 cm around each tube, and accordingly parameters such as time spent in the tube zones and number of entries inside each tube zone was recorded. For each animal, cagemate tube opening behavior across the three trials in which a cagemate tube was presented (Cagemate versus Empty, ×2, and Cagemate versus Chocolate, ×1) was classified into two categories: “Cagemate Openers,” corresponding to mice that successfully opened the cagemate-containing tube in at least one of the three trials, and “Non-Openers,” corresponding to mice that failed to open the cagemate tube in any of the three trials (Figures 3A–3F).

#### Score for empathy-related behavior

To assess the empathy-related profile of nicotinic mutants as compared to WT controls, a score was calculated according to two factors: namely, Cagemate Prioritization (CP) which allocated a score between −3 and 3 to the mice depending on whether the cagemate tube (+1), the chocolate tube (−1) or the empty tube (−1) was opened first during trials cagemate versus empty and cagemate versus chocolate. The second factor was social-contact positivity (SCP) and allocated a score between −3 and 3 to mice depending on whether interacting (+1), disinterested (0) or aggressive (−1) behavior was displayed toward the cagemate after the test. The interaction type was categorized in real-time during the test by two experimenters, working independently and blind to genotype: Interactive behavior was attributed when a minimum of 10 sniffings where observed, on the contrary when less than 10 sniffings were observed and the mice spent most of the interaction time at opposite ends of the cage, the interaction was considered as disinterested. Aggressive behavior was considered when 2 or more clearly identifiable antagonistic events (chasings and/or biting) were recorded between the test mouse and the cagemate, typically accompanied by ultrasonic vocalizations from the freed cagemate and active mounting or tail rattling from the aggressor. In the latter case, the session was immediately stopped. All sessions were additionally re-scored offline from video recordings by a third rater (NH), blind to genotype and sex and to the original real-time categorization. Inter-rater agreement between raters was assessed using Cohen’s κ (κ = 0.75).^102^ Representative videos illustrating each post-release profile are provided as Supplementary Videos: aggressive (Video S4), disinterested (Video S5), and interactive (Video S6).

Score formula:

$${SCORE}_{\;empathy-related}=\frac{CP+SCP}{2}$$

#### Three chamber sociability test

A separate cohort of 23 male (WT, *n* = 7; α5KI, *n* = 8; and α5KO, *n* = 8) and 28 female (WT, *n* = 11; α5KI, *n* = 8, and α5KO, *n* = 9) mice were tested in a three-chamber sociality paradigm adapted from Rein et al.,.^103^ The apparatus consisted of a rectangular box divided into three compartments: Two larger side chambers, each containing an inverted wire cup, and a smaller central chamber. Testing occurred across a single day. In the morning, subjects were habituated to the apparatus for 10 min, with free access to all compartments. In the afternoon, mice were confined to the central chamber for 5 min before the start of testing. An age- and sex-matched conspecific (social stimulus) was placed under one of the wire cups, while the cup in the opposite chamber remained empty. The doors for each of the compartments were then lifted, allowing the subject to freely explore all compartments for 10 min. Time spent (seconds) in each compartment was recorded using Anymaze behavioral tracking software. A sociability index was computed with the following formulas

$$Sociability\;Index=\frac{Social\;cpt\;time-Non\;social\;cpt\;time}{Social\;cpt\;time-Non\;social\;cpt\;time}$$

### Quantification and statistical analysis

Statistical analyses were performed using GraphPad Prism and StatView. No animals were excluded from analysis. All tests were two-tailed, with significance set at α = 0.05. Normality of data distribution was assessed using the Kolmogorov-Smirnov test prior to selecting parametric or non-parametric approaches.

#### Parametric analyses

Continuous behavioral measures following a normal distribution were analyzed using one-way, two-way, or three-way ANOVA with genotype and/or sex as between-subject factors, or repeated-measures ANOVA (RM-ANOVA) with trial or time as the within-subject factor (e.g., time in cylinder zones across habituation and test trials, Figures 1A and 1D; cumulated time in the SD zone across 30-s bins; Figures 1C and 1F; self-freeing and chocolate-tube opening latencies across trials; Figure 3; tube preference during the competition trial; Figure 4; post-release interaction scores; Figure 5A). Where Mauchly’s test indicated a violation of sphericity, Greenhouse-Geisser-corrected degrees of freedom are reported. Significant main or interaction effects were followed by Bonferroni/Dunn or Tukey’s post-hoc tests for pairwise comparisons between genotypes. Paired-sample (e.g., SD vs. ND zone time or sniffing counts within each group, Figures 1B, 1E, 2A and 2C) and one-sample t-tests (e.g., sociability index compared to zero, Figure S5) were used where appropriate. Effect sizes are reported alongside their 95% confidence intervals. For ANOVA effects, partial η^2^ was calculated as (df_effect × F)/(df_effect × F + df_error). For paired and one-sample t-tests, Cohen’s d was computed as t/√n, where n is the number of within-subject observations. For Mann-Whitney tests, the rank-biserial correlation was computed as r = z/√N. For Kruskal–Wallis tests, η^2^_H was computed as H/(*n* − 1). Effect sizes and their 95% confidence intervals were computed in Python using the Pingouin package.

#### Non-parametric and categorical analyses

Frequencies of categorical behavioral profiles (e.g., discriminator vs. non-discriminator classification, Figures 2B and 2D) were compared using Chi-square (χ^2^) tests, with Cohen’s w reported as the effect size; Fisher’s exact test (or, for contingency tables with more than two categories, the Fisher-Freeman-Halton exact test) was used instead of χ^2^ when expected cell counts were small (<5). For the cagemate tube-opening profile (Figures 3A–3F), animals were classified as “Openers” (at least one successful cagemate-tube-opening event across the three applicable trials) or “Non-Openers” (no successful opening event); given small expected cell counts, the proportion of openers in each sex-by-genotype group was compared against the chance level of 50% using an exact binomial test rather than a between-group contingency test. For the trial-by-trial cagemate-tube and empty-tube opening frequency data (Figures 3G–3J), the Fisher-Freeman-Halton exact test was used given small expected cell counts, particularly in the α5KO group (males *n* = 8, females *n* = 5). Empathy-related scores (Figure 5B), which combine ordinal components, were compared across the three genotypes using the non-parametric Kruskal-Wallis test (η^2^H reported as effect size), and between two groups (e.g., sex effect; comparison of WT subgroups by line of origin) using the non-parametric Mann-Whitney U test (r reported as effect size, calculated as Z/√n). Spearman’s rank correlation (ρ) was used to assess the relationship between the two components of the composite empathy-related score (cagemate prioritization and social-contact positivity).

#### Primary and exploratory outcomes

Zone occupancy time, the SD/cagemate preference score, and tube-opening frequencies (cagemate prioritization, CP) constituted our primary, prespecified outcome measures. Sniffing counts were added subsequently as a complementary measure, providing a more behaviorally specific readout of investigative interest to corroborate the zone-occupancy findings, and are reported alongside the primary outcomes but were not themselves prespecified. Post-release interaction category profiling (interactive/disinterested/aggressive), and the resulting Social-Contact Positivity (SCP) measure, were developed subsequently, based on behavioral patterns identified during scoring, and are reported as exploratory, hypothesis-generating analyses. The composite empathy-related score, which combines the prespecified CP component with the exploratory SCP component, was likewise developed during the analysis phase rather than prespecified in its final form, and is presented as an exploratory measure integrating both dimensions of the behavioral response. None of the exploratory measures were corrected for multiple comparisons.

## Supplemental information

Document S1. Figures S1–S5 and Table S1

Data S1. Raw data from the three-chamber sociability test, related to Figure S5

Data S2. Raw data from the affective state discrimination test, related to Figures 1, 2, S1, S2, S3

Data S3. Raw data from the RTT, related to Figures 3, 4, and 5, S4, and Table S1
