# Supplementary material for: Alteration of empathy-related behaviors in two strains of mice expressing mutations of the α5 cholinergic nicotinic receptor subunit
Source: iScience. 2026 Oct 17;29(11):117950. doi: 10.1016/j.isci.2026.117950 (PMC13695379; doi:10.1016/j.isci.2026.117950)
Supplement: Supplementary file 1 [file mmc1.pdf]

iScience, Volume *29*

Supplemental information

Alteration of empathy-related behaviors in two strains of mice expressing mutations of the α5 cholinergic nicotinic receptor subunit

Léa Tochon, Cécile Pagèze, Nadia Henkous, Jean-Louis Guillou, and Vincent David

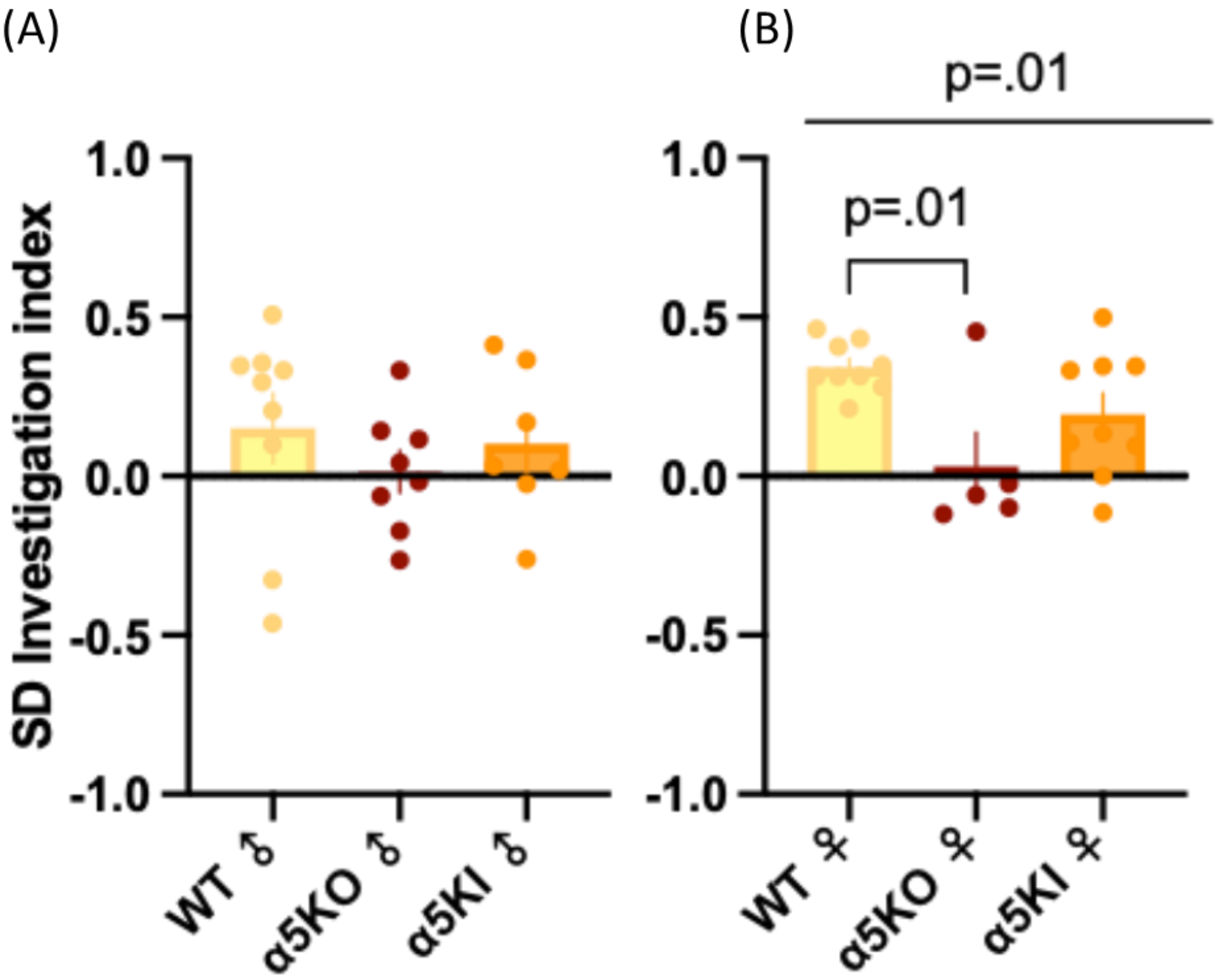

Figure S1. Impairment of exploratory behavior towards a stressed demonstrator in α5 nicotinic mutants. Stressed Demonstrator Investigation Index (SD sniffing – ND sniffing)/ Total sniffings), normalized SD preference score in males **(A)** and females **(B).**

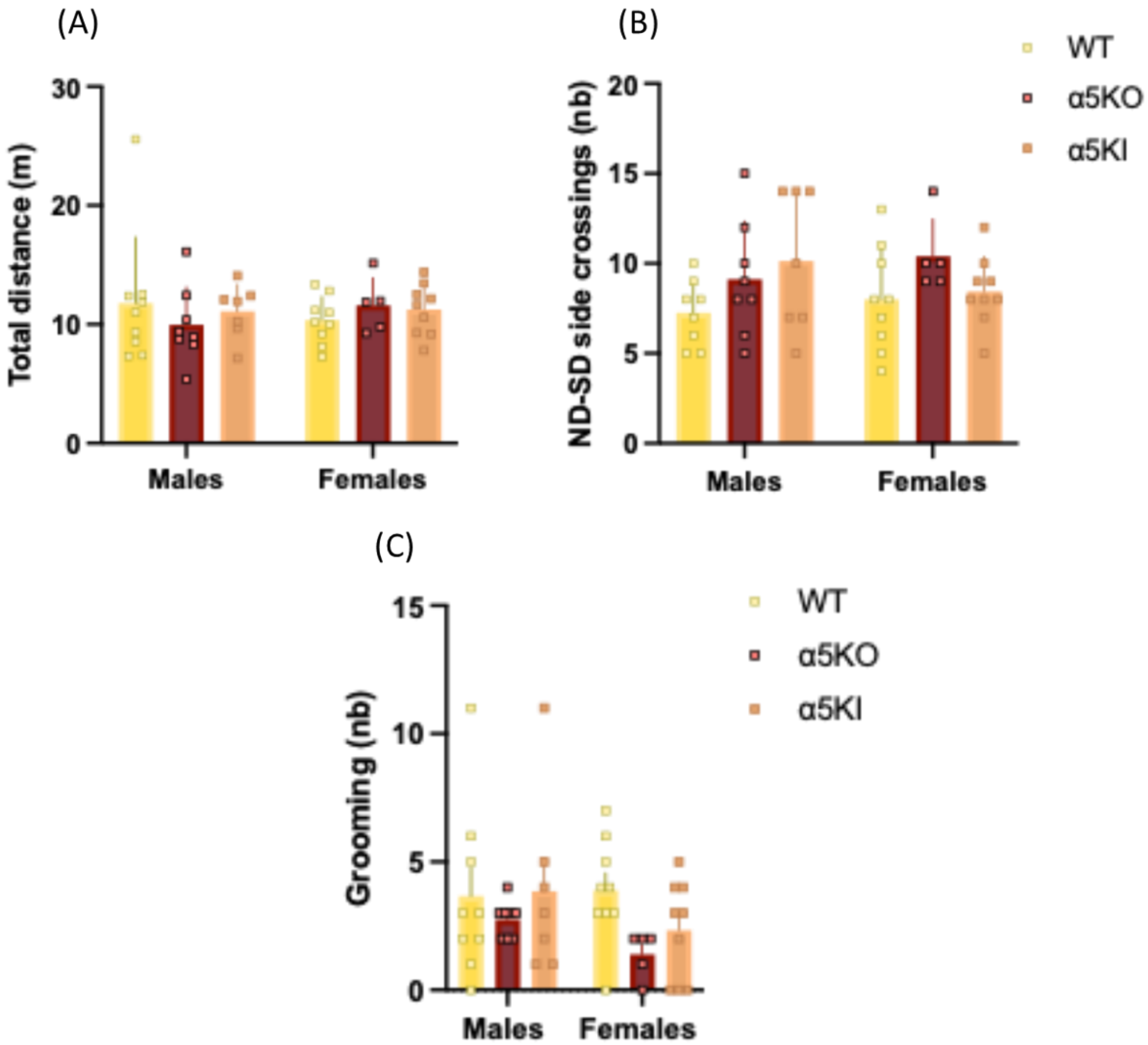

Figure S2. α5 mutants do not display locomotor differences or spatial asymmetry in the ASDT. (A) Bar graph of total distance travelled in the apparatus during the 6-minute duration of the test in WT, α5KO and α5KI mice **(B)** Bar graph of side crossings between the two demonstrator zones (SD-ND) in WT, α5KO and α5KI mice. **(C)** Bar graph of grooming events recorded during the 6-minute test in WT, α5KO and α5KI mice. One way ANOVA were run to compare groups, there is no significant difference.

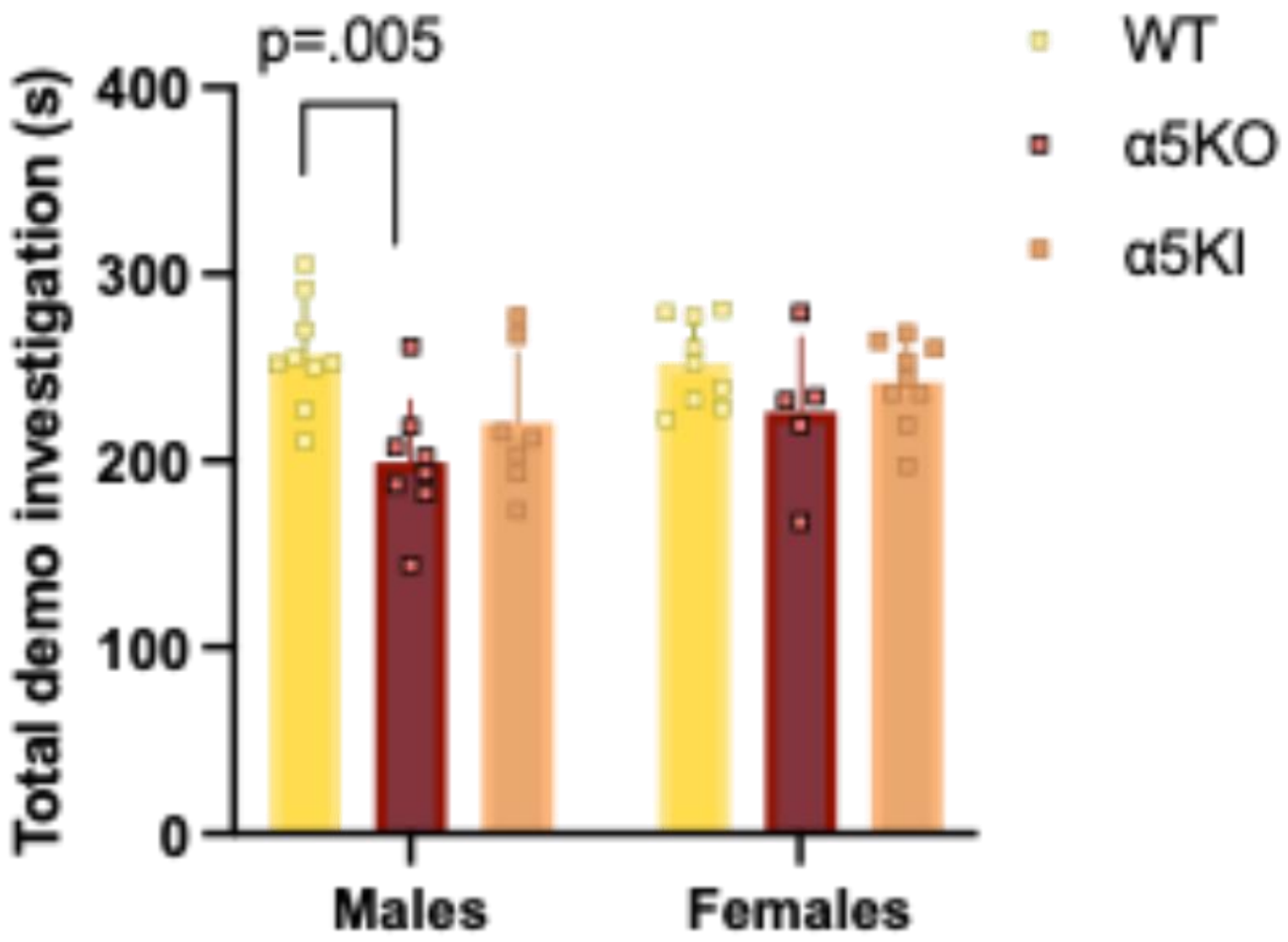

Figure S3. Male but not female α5KO spend less time investigation demonstrators compared to WT controls (A) Bar graph of total time spent investigating demonstrators (in seconds) in males (left) and females (right). Male genotype effect $p<.01$, Tukey's post-hoc: WT vs α5KO, $p=.005$.

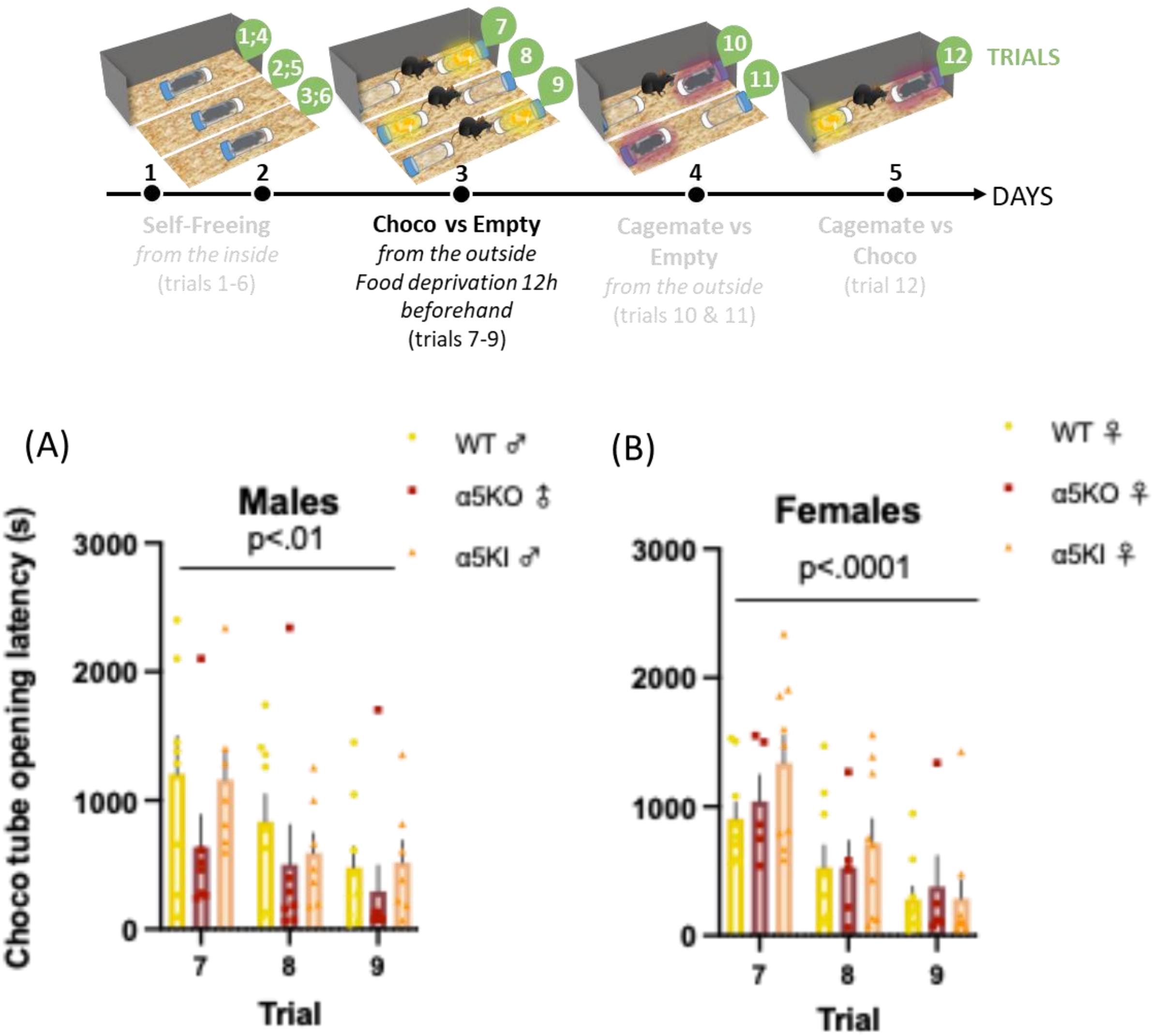

Figure S4. α5 mutants display an intact ability to learn how to open a chocolate containing tube. Bar graph of opening latency of the chocolate containing tube in the chocolate versus empty tube trials (trial 7,8,9 of training day 3) in males **(A)** and females **(B).** Groups were compared with One-Way ANOVAs.

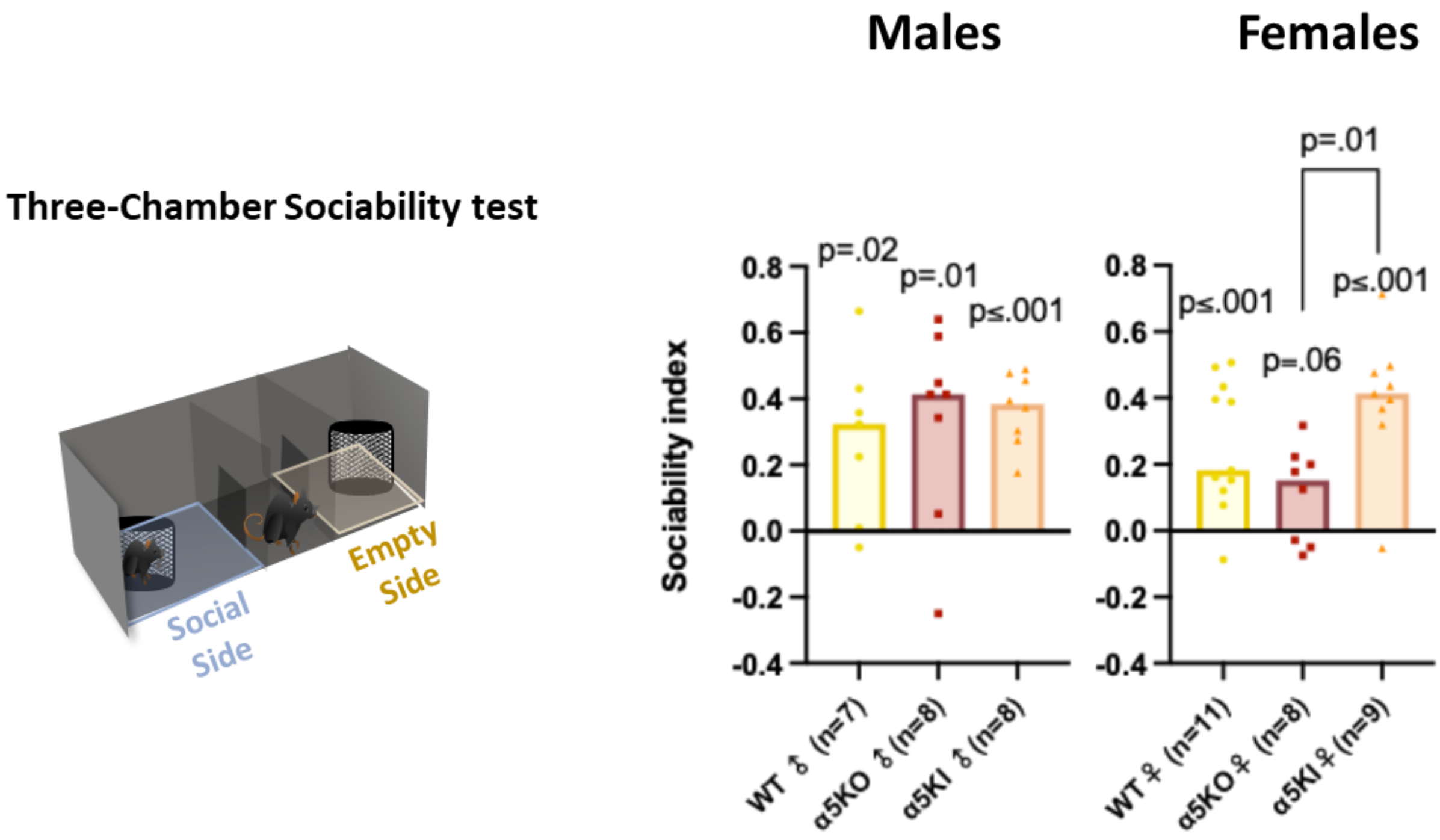

Figure S5. α5 mutants display intact social preference in the three-chamber sociability test. Bar graph representing the sociability index = (Time spent in social cpt – Time spent in non-social cpt)/(Time spent in social cpt + Time spent in non-social cpt) for males, p values above each bar graph represent the statistics for the one sample t-test against theoretical value 0, in males **(A)** and females **(B);** genotype effect $p<.05$, tukey's post-hoc: α5KI vs α5KO, $p=.01$.

Score variant	Sex	Genotype effect	Key pairwise contrast
Original (CP+SCP)/2	F	H=7.96, p=.018	—
	M	H=4.15, p=.12 (ns)	—
SCP ×2: (CP+2×SCP)/3	F	H=8.18, p=.017	KO vs KI: p=.005
	M	H=5.84, p=.054 (trend)	KO vs KI: p=.030
CP alone	F	H=6.73, p=.035	KO vs KI: p=.030
	M	H=0.02, p=.993 (ns)	—

Table S1. Robustness of the empathy-related composite score under alternative weighting and scoring schemes, related to Figure 5. Kruskal-Wallis tests of genotype effects on the empathy-related score, computed under the original scoring formula (SCORE = (CP + SCP)/2) and two alternative specifications: (i) increased weighting of the aggression/post-release component (SCORE = (CP + 2×SCP)/3), and (ii) cagemate prioritization (CP) considered alone, independently of post-release social behavior. Genotype and sex effects, together with the strongest pairwise contrast for each sex, are reported for each scoring scheme. Genotype and sex effects were preserved, and in some cases strengthened, when the aggression component was upweighted. In contrast, using CP alone abolished the genotype effect in males (H=0.02, p=.993) and the overall sex effect (H=2.48, p=.116), reflecting the fact that α5KO males opened the cagemate tube at rates comparable to WT but subsequently displayed aggressive post-release behavior. This dissociation between tube-opening and post-release social quality supports the rationale for combining both components into a single composite score. n.s., not significant.
